# Human leukocyte antigen alleles associate with COVID-19 vaccine immunogenicity and risk of breakthrough infection

**DOI:** 10.1038/s41591-022-02078-6

**Published:** 2022-10-13

**Authors:** Alexander J. Mentzer, Daniel O’Connor, Sagida Bibi, Irina Chelysheva, Elizabeth A. Clutterbuck, Tesfaye Demissie, Tanya Dinesh, Nick J. Edwards, Sally Felle, Shuo Feng, Amy L. Flaxman, Eleanor Karp-Tatham, Grace Li, Xinxue Liu, Natalie Marchevsky, Leila Godfrey, Rebecca Makinson, Maireid B. Bull, Jamie Fowler, Bana Alamad, Tomas Malinauskas, Amanda Y. Chong, Katherine Sanders, Robert H. Shaw, Merryn Voysey, Oxford COVID Vaccine Trial Genetics Study Team Group, Ana Cavey, Angela Minassian, Arabella Stuart, Baktash Khozoee, Brama Hanumunthadu, Brian Angus, Catherine C. Smith, Iain Turnbull, Jonathan Kwok, Katherine R. W. Emary, Liliana Cifuentes, Maheshi N. Ramasamy, Paola Cicconi, Adam Finn, Alastair C. McGregor, Andrea M. Collins, Andrew Smith, Anna L. Goodman, Christopher A. Green, Christopher J. A. Duncan, Christopher J. A. Williams, Daniela M. Ferreira, David P. J. Turner, Emma C. Thomson, Helen Hill, Katrina Pollock, Mark Toshner, Patrick J. Lillie, Paul Heath, Rajeka Lazarus, Rebecca K. Sutherland, Ruth O. Payne, Saul N. Faust, Tom Darton, Vincenzo Libri, Rachel Anslow, Samuel Provtsgaard-Morys, Thomas Hart, Amy Beveridge, Syed Adlou, Matthew D. Snape, Andrew J. Pollard, Teresa Lambe, Julian C. Knight

**Affiliations:** 1 Wellcome Centre for Human Genetics, Nuffield Department of Medicine, University of Oxford, Oxford, UK; 2 Oxford Vaccine Group, Department of Paediatrics, University of Oxford, Oxford, UK; 3 NIHR Oxford Biomedical Research Centre and Oxford University Hospitals NHS Foundation Trust, Oxford, UK; 4 The Jenner Institute, Nuffield Department of Medicine, University of Oxford, Oxford, UK; 5 Chinese Academy of Medical Science (CAMS) Oxford Institute, University of Oxford, Oxford, UK; 6 Division of Structural Biology, Wellcome Centre for Human Genetics, University of Oxford, Oxford, UK; 7 Nuffield Department of Clinical Neurosciences, University of Oxford, Oxford, UK; 8 Department of Biochemistry, University of Oxford, Oxford, UK; 9 Nuffield Department of Population Health, University of Oxford, Oxford, UK; 10 Nuffield Department of Medicine, University of Oxford, Oxford, UK; 11 Kennedy Institute of Rheumatology, Nuffield Department of Orthopaedics, University of Oxford, Oxford, UK; 12 School of Population Health Sciences, University of Bristol and University Hospitals Bristol and Weston NHS Foundation Trust, Bristol, UK; 13 London Northwest University Healthcare, Harrow, UK; 14 Department of Clinical Sciences, Liverpool School of Tropical Medicine, Liverpool, UK; 15 College of Medical, Veterinary & Life Sciences, Glasgow Dental Hospital & School, University of Glasgow, Glasgow, UK; 16 Guy’s and St. Thomas’ NHS Foundation Trust, London, UK; 17 MRC Clinical Trials Unit at UCL, London, UK; 18 NIHR/Wellcome Trust Clinical Research Facility, University Hospitals Birmingham NHS Foundation Trust, Birmingham, UK; 19 Department of Infection and Tropical Medicine, Newcastle upon Tyne Hospitals NHS Foundation Trust, Newcastle, UK; 20 NIHR Newcastle Clinical Research Facility, Newcastle upon Tyne Hospitals NHS Foundation Trust, Newcastle, UK; 21 Translational and Clinical Research Institute, Immunity and Inflammation Theme, Newcastle University, Newcastle, UK; 22 Public Health Wales, Cardiff, Wales and Aneurin Bevan University Health Board, Wales, Cardiff, UK; 23 School of Life Sciences, University of Nottingham and Nottingham University Hospitals NHS Trust, Nottingham, UK; 24 MRC - University of Glasgow Centre for Virus Research & Department of Infectious Diseases, Queen Elizabeth University Hospital, Glasgow, UK; 25 NIHR Imperial Clinical Research Facility and NIHR Imperial Biomedical Research Centre, London, UK; 26 Department of Medicine, Cambridge Heart Lung Research Institute, University of Cambridge, Cambridge, UK; 27 Hull University Teaching Hospitals NHS Trust, Hull, UK; 28 St. George’s Vaccine Institute, University of London, London, UK; 29 Department of Infection, Sheffield Teaching Hospitals NHS Foundation Trust, Sheffield, UK; 30 Severn Pathology, North Bristol NHS Trust, Bristol, UK; 31 Clinical Infection Research Group, Regional Infectious Diseases Unit, NHS Lothian, Edinburgh, UK; 32 Department of Infection, Immunity and Cardiovascular Diseases, University of Sheffield, Sheffield, UK; 33 NIHR Southampton Clinical Research Facility and Biomedical Research Centre, University Hospital Southampton NHS Foundation Trust, Southampton, UK; 34 Faculty of Medicine and Institute for Life Sciences, University of Southampton, Southampton, UK; 35 NIHR UCLH Clinical Research Facility and NIHR UCLH Biomedical Research Centre, London, UK

**Keywords:** Genetics research, Preventive medicine

## Abstract

Severe acute respiratory syndrome coronavirus 2 (SARS-CoV-2) vaccine immunogenicity varies between individuals, and immune responses correlate with vaccine efficacy. Using data from 1,076 participants enrolled in ChAdOx1 nCov-19 vaccine efficacy trials in the United Kingdom, we found that inter-individual variation in normalized antibody responses against SARS-CoV-2 spike and its receptor-binding domain (RBD) at 28 days after first vaccination shows genome-wide significant association with major histocompatibility complex (MHC) class II alleles. The most statistically significant association with higher levels of anti-RBD antibody was HLA-DQB1*06 (*P* = 3.2 × 10^−9^), which we replicated in 1,677 additional vaccinees. Individuals carrying HLA-DQB1*06 alleles were less likely to experience PCR-confirmed breakthrough infection during the ancestral SARS-CoV-2 virus and subsequent Alpha variant waves compared to non-carriers (hazard ratio = 0.63, 0.42–0.93, *P* = 0.02). We identified a distinct spike-derived peptide that is predicted to bind differentially to HLA-DQB1*06 compared to other similar alleles, and we found evidence of increased spike-specific memory B cell responses in HLA-DQB1*06 carriers at 84 days after first vaccination. Our results demonstrate association of HLA type with Coronavirus Disease 2019 (COVID-19) vaccine antibody response and risk of breakthrough infection, with implications for future vaccine design and implementation.

## Abstract (web summary)

Variation in antibody levels elicited by the COVID-19 vaccine ChAdOx1 nCov-19 is linked to specific major histocompatibility complex class II alleles, providing insight into the breadth of immune response among vaccinated individuals.

## Main

Since its emergence in late 2019, SARS-CoV-2 has caused a global pandemic with estimates of between 6.5 and 15 million deaths up to September 2022 (refs. ^1,2^). Vaccines targeting, predominantly, the spike antigen of SARS-CoV-2 have demonstrated high efficacy against severe disease in phase 3 trials, eliciting high levels of binding and neutralizing antibodies, as well as T cell responses, with over 12 billion doses administered worldwide^1^. Two of the earliest developed vaccines, BNT162b2 (Pfizer-BioNTech)^3^ and ChAdOx1 nCoV-19 (AZD1222, Oxford-AstraZeneca)^4^, are estimated to have population effectiveness against a positive polymerase chain reaction (PCR) test for the earliest variants of SARS-CoV-2 of 79% and 80%, respectively, when assessed at least 21 days after the second dose of vaccination in a community-based household survey from the United Kingdom (UK) (1 December 2020 to 8 May 2021)^5^, together with 88–91% effectiveness against hospital admission for COVID-19 (ref. ^5^), although lower effectiveness is reported with more recent variants of concern^6^. Despite the success of vaccines at reducing mortality and morbidity in the population, with effectiveness against severe disease and hospitalization currently remaining high, vaccine breakthrough infections, although predominantly mild, are increasingly reported^7–9^.

Considerable variation in immune responses, including antibody levels and T cell responses, has been reported among vaccinated individuals^10^. Neutralizing antibody levels show association with vaccine efficacy in animal challenge studies^11^ and humans^12,13^, and risk of symptomatic COVID-19 has been shown to reduce with increasing levels of both anti-spike (anti-S IgG) and antibodies against RBD antigenic sites on the viral spike (anti-RBD IgG) after vaccination with ChAdOx1 nCoV-19 (ref. ^13^). The reasons for inter-individual variation in total or neutralizing antibody responses are incompletely understood^10,14^. Community-based surveys have provided some epidemiological insight into this question among individuals with no prior history of SARS-CoV-2 infection in the UK general population: a low anti-S IgG antibody responder group after vaccination was identified and found to be more commonly male, elderly (over 75 years of age) and with long-term health conditions^10^.

We sought to investigate the contribution of genetic factors to the observed variation in response to vaccination with ChAdOx1 nCoV-19. Antibody responses after vaccination show evidence of heritability^15^, with genetic variation in HLA within the MHC on chromosome 6 (position p21.3) associated with responses to hepatitis B^16–19^, tetanus^20^ and measles^21,22^ vaccines. For these infections, the relevance for vaccine failure has not been robustly demonstrated^23,24^. To date, genetic studies in COVID-19 have focused on risk of severe disease, with replicated associations implicating antiviral defense mechanisms (notably involving interferon signaling), mediators of inflammatory organ damage, leucocyte differentiation and blood type antigen secretor status but limited evidence to date for HLA^25–27^. In this study, we used data from five clinical trials of ChAdOx1 nCoV-19 to demonstrate association of HLA-DQB1*06 with higher antibody responses against the RBD of spike antigen and lower risk of breakthrough infections, which we propose involves altered HLA peptide binding influencing memory B cell responses.

## Results

### Genome-wide association study of antibody responses 28 days after ChAdOx1 nCoV-19 vaccination

**Fig. 1:**
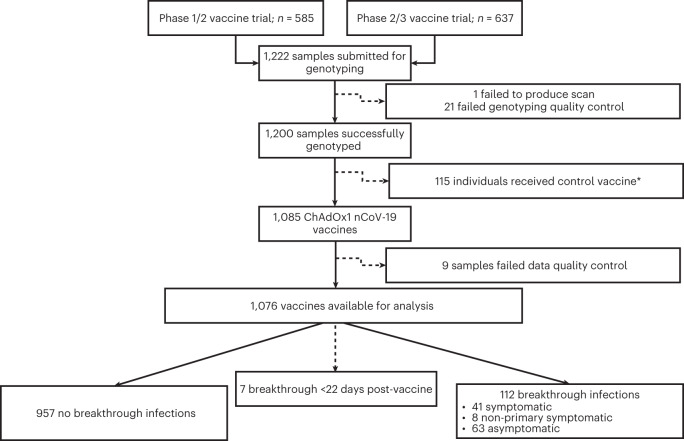
Flow diagram of participants selected for analysis from the phase 1/2 (COV001) and phase 2/3 (COV002) vaccine trials. For breakthrough infections, symptomatic individuals had primary symptoms of COVID-19 (cough, shortness of breath, fever, anosmia or ageusia); if they presented with symptoms other than the five primary COVID-19 symptoms, they were categorized as non-primary symptomatic cases. *Samples selected to maintain investigator blinding during sample selection.

**Fig. 2:**
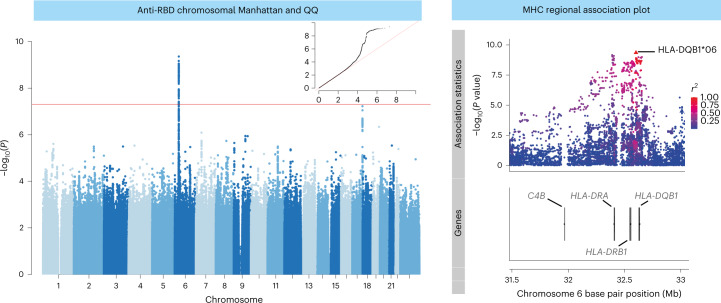
Genome-wide and MHC SNP associations with RBD antibody level in 1,076 ChAdOx1 nCoV-19 vaccine recipients from the COV001 and COV002 vaccine trials. The association results for all tested autosomal and X chromosome variants with linear regression in a mixed-model framework are shown on the left in a Manhattan plot, with the red horizontal line representing the nominal threshold for GWAS significance (*P* = 5 × 10^−8^) to account for the multiple tests performed. The QQ plot in the inset of the Manhattan plot on the left of the figure with expected *P* values shown on the *x* axis and observed on the *y* axis. A magnified view of a portion of the MHC locus is shown on the right of the figure. All points represent SNPs or HLA alleles positioned by build 37 of the human genome coordinates and colored on the right by linkage disequilibrium (*r*^2^), with relevant gene coordinates provided in the lower panel.

**Extended Data Fig. 1:**
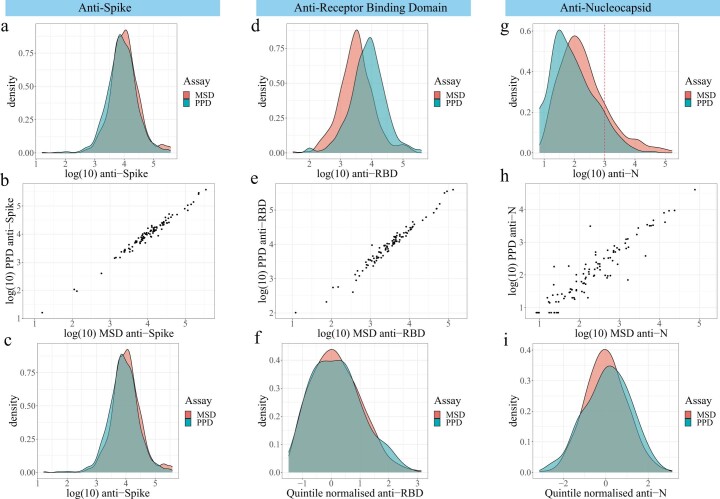
Immunoassay distributions. IgG antibody responses against SARS-CoV-2 spike (S, panels **a-c**), receptor binding domain (RBD, panels **d-f**) and nucleocapsid (N, panels **g-i**)) were measured for COV001 and COV002 participants in two laboratories: MSD and PPD. For 97 individuals, measures were available from both laboratories. The density distributions of measures from each laboratory for each antigen (panels **a**, **d** and **g**) and the correlation between intra-individual measures (panels **b** rho 0.98, **e** rho 0.98, and **h** rho 0.90) were inspected. If the density distributions overlapped and the intra-individual correlation (r^2^) was greater than 0.7, the original distributions were taken forward for analysis (as for S, panel **c**). However if r^2^ was less than 0.7, the quintile normalised intra-laboratory measures were taken forwards for analysis (panels **f** and **i**).

**Extended Data Fig. 2:**
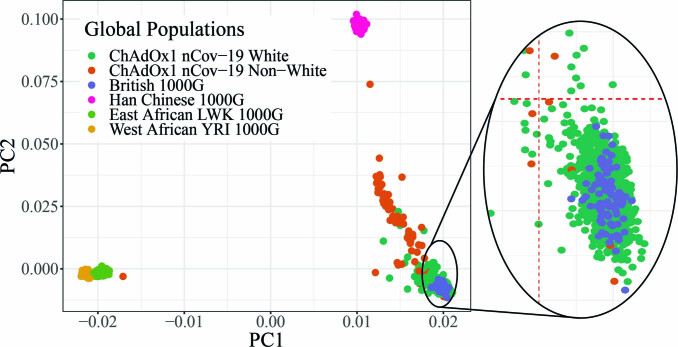
Principal components of genotyped individuals from COV001 and COV002 alongside individuals from 1000 Genomes (1000 G) European, Asian and African populations. The European cluster is magnified in the inset to demonstrate effective overlap between the majority of self-reported White ChAdOx1 nCoV-19 individuals and the British (from England and Scotland) 1000 G individuals. Red dashed lines represent PC1 and PC2 thresholds to define a specific European cluster.

**Extended Data Fig. 3:**
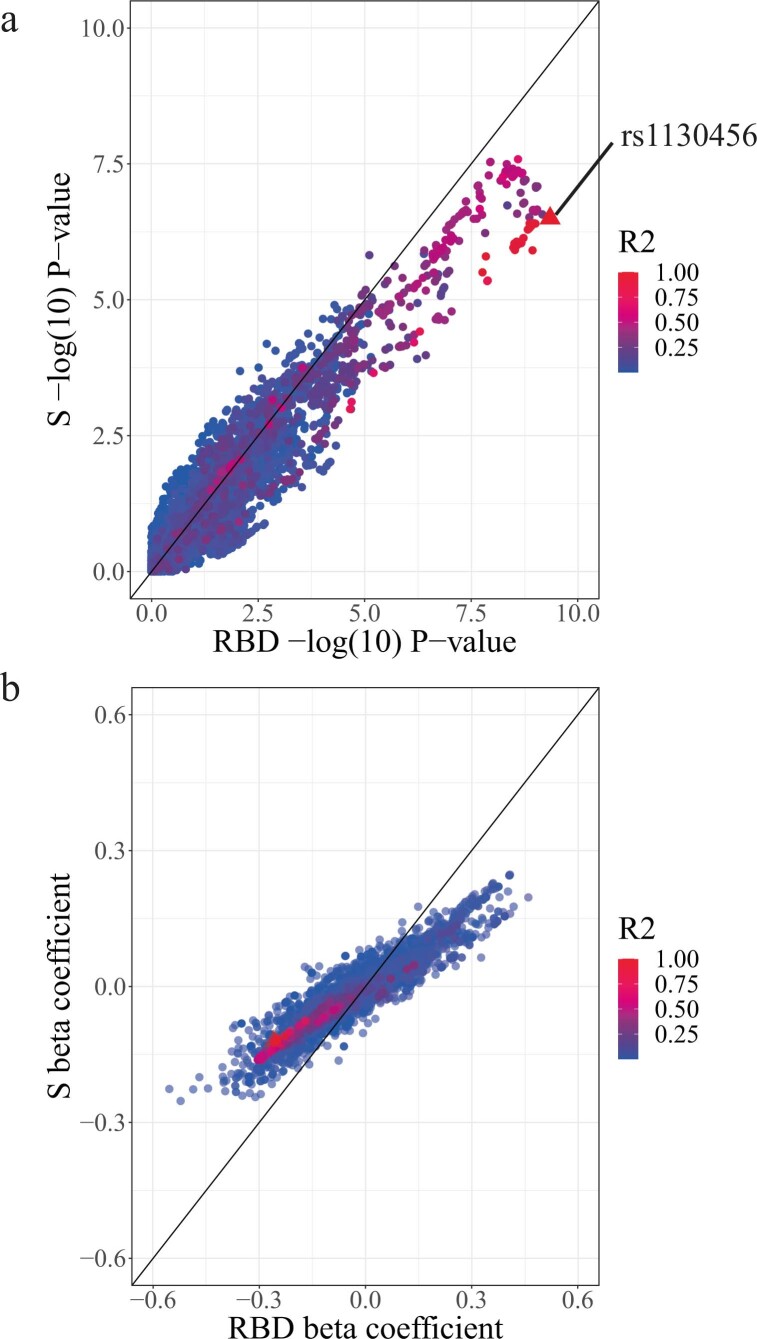
Correlation in genetic architecture between association statistics for anti-Spike and anti-RBD in individuals vaccinated with ChAdOx nCov-19 in COV001 and COV002. Comparison of –log(10) P-values (**a**) and beta coefficients (**b**) for genotyped and imputed variants tested using linear regression within 4 Mb window of top associated variant associated with RBD (rs1130456). Points are coloured by linkage disequilibrium (r^2^) with rs1130456 and the index variant is shaped as a triangle compared to all other variants as circles.

We hypothesized that genetic factors contribute to inter-individual variation in COVID-19 vaccine responses. To investigate this, we first performed a discovery analysis testing for genetic association with vaccine responses in participants enrolled in the phase 1/2 (COV001) and phase 2/3 (COV002) randomized single-blind ChAdOx1 nCov-19 (AZD1222) vaccine efficacy trials, conducted within the UK, and in whom humoral immune responses were measured after vaccination. Figure 1 summarizes participant inclusion. DNA from 1,222 ChAdOx1 nCov-19 trial participants was genotyped on the Affymetrix AxiomTM HGCoV2 1 array. After quality control (Methods), 667,496 variants in 1,190 individuals were available for single-nucleotide polymorphism (SNP) variant imputation (Supplementary Fig. 1). After imputation, 9,325,058 high-quality SNPs were tested for association with normalized antibody responses against spike and RBD (Extended Data Fig. 1) in 1,076 of the 1,190 genotyped individuals who had received ChAdOx1 nCoV-19 vaccine, with antibody measures available at 28 days after first vaccination (baseline demographics are shown in Table 1). We performed the association analysis adjusting for age, sex, prior SARS-CoV-2 exposure based on anti-nucleocapsid (anti-N) IgG concentrations (*n* = 128, 11.9%) and antibody assay type (all as fixed-effect covariates) for all individuals irrespective of ancestry (Extended Data Fig. 2), including a genetic relatedness matrix (GRM) as a random effect covariate. The mixed model regression analysis revealed genome-wide significant associations (*P* < 5 × 10^−8^) for both anti-spike (index variant rs9271374, *P* = 2.6 × 10^−8^, beta= −0.14 and s.e. = 0.03) and anti-RBD (rs1130456, *P* = 4.4 × 10^−10^, beta = −0.26 and s.e. = 0.04) IgG antibody levels. rs9271374 and rs1130456 are SNPs located within 10 kilobases (kb) of *HLA-DQ* genes (Fig. 2) and in linkage disequilibrium within our multi-ancestry cohort (*r*^2^ = 0.65). The distribution of *P* values (Extended Data Fig. 3a) and beta coefficients (Extended Data Fig. 3b) for all genotyped and imputed variants across this locus show a clear correlation in genetic architecture between these two antibody responses (Spearman’s rho coefficient 0.90 and 0.93 for *P* values and beta coefficients, respectively) correlated through linkage disequilibrium (measured through *r*^2^).

**Table 1:** Baseline characteristics for 1,076 ChAdOx1 nCoV-19 participants in the phase 1/2 (COV001) and phase 2/3 (COV002) trials who received ChAdOx1 nCoV-19 vaccine with genotype data available and for the 1,069 individuals who either experienced a breakthrough infection after 21 days or who did not experience a breakthrough up to time of censoring

Characteristic (range where applicable)	Genotyped cohort (ChAdOx1 vaccinated) *n* = 1,076	No reported breakthrough infection (*n* = 957)	Breakthrough infection reported (*n* = 112)	Evidence of difference between breakthrough groups (*P*)
Age at recruitment	37.0 (30.0–47.0)	37.0 (30.0–47.0)	37.0 (28.8–48.0)	NS
18–55 years, number (%)	1076 (100)	957 (100)	112 (100)	
Missing, number (%)	0 (0)	0 (0)	0 (0)	NS
Sex, number (%)				
Female	572 (53.2)	514 (53.7)	56 (50.0)	
Male	504 (46.8)	443 (46.3)	56 (50.0)	NS
BMI				
Median (IQR)	24.7 (22.6–27.5)	24.7 (22.5–27.5)	25.2 (22.7–28.3)	NS
Ethnic group, number (%)				
White	974 (90.5)	865 (90.4)	102 (91.1)	
Asian	50 (4.7)	44 (4.6)	6 (5.3)	
Black	10 (0.9)	9 (0.9)	1 (0.9)	
Mixed	29 (2.7)	26 (2.7)	3 (2.7)	
Other	13 (1.2)	13 (1.4)	0 (0)	
Not reported/missing	0 (0)	0 (0)	0 (0)	NS
Health and social care setting workers (HCWs), number (%)				
Not HCW	590 (54.8)	539 (56.3)	48 (42.9)	
HCW unknown COVID contacts	101 (9.4)	91 (9.5)	10 (8.9)	
HCW with ≤1 COVID contacts	267 (24.8)	226 (23.6)	40 (35.7)	
HCW with >1 COVID contacts	118 (11.0)	101 (10.6)	14 (12.5)	0.02
Comorbidities, number (%)				
Cardiovascular	35 (3.3)	29 (3.0)	6 (5.2)	NS
Respiratory	73 (6.8)	59 (6.1)	14 (12.5)	0.02
Diabetes	9 (0.8)	7 (0.7)	2 (1.8)	NS
Interval between first and second vaccine, number (%)				
<6 weeks	12 (1.1)	11 (1.1)	1 (0.9)	
6–8 weeks	151 (14.0)	133 (13.9)	17 (15.2)	
9–11 weeks	257 (23.9)	220 (23.0)	33 (29.5)	
≥12 weeks	556 (51.7)	501 (52.4)	53 (47.3)	
None	100 (9.3)	92 (9.6)	8 (7.1)	NS
Vaccines received before infection occurring, number (%)				
SD	499 (46.4)	463 (48.4)	35 (31.3)	
SD/SD	400 (37.2)	342 (35.7)	54 (48.2)	
LD/SD	167 (15.5)	143 (15.0)	22 (19.6)	
SD/LD	10 (0.9)	9 (0.9)	1 (0.9)	0.005
HLA carrier, number (%)				
HLA-DQB1*06	474 (44.1)	436 (45.6)	38 (33.9)	0.02
DRB1-71E/R	881 (81.9)	784 (81.9)	92 (82.1)	NS
HLA status, number (%)				
Carrying DRB1-71E/R with no DQB1*06	532 (49.4)	464 (48.5)	63 (56.3)	
Remainder	419 (38.9)	377 (39.4)	40 (35.7)	
Carrying DQB1*06 with no DRB1-71E/R	125 (11.6)	116 (12.1)	9 (8.0)	NS

Seven individuals were not included in the breakthrough analysis as they experienced breakthrough infection within 22 days of receiving their first vaccine. Statistical differences were tested across groups using chi-squared tests or Fisher exact tests if any group contained fewer than five observations. NS, not significant.

**Extended Data Fig. 4:**
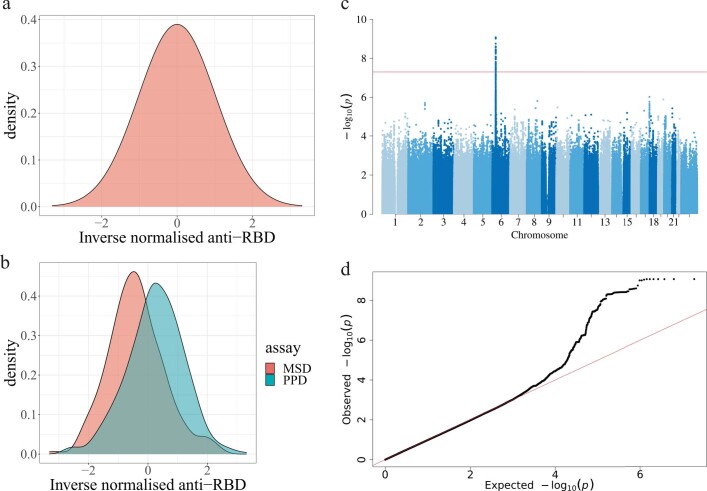
Testing effect of non-normality of antibody distributions on HLA association signals in COV001 and COV002 participants. The final RBD antibody levels were inverse normalised across all individuals with genotype data available to produce a normal phenotype distribution (**a**), with preserved distributions when considered stratified by assay (**b**). The association signal across the MHC when tested for using linear mixed-model regression was still observed with the genome-wide association statistics presented in Manhattan (with the GWAS level of significance represented by the horizontal red line) (**c**) and QQ plots (**d**).

**Extended Data Fig. 5:**
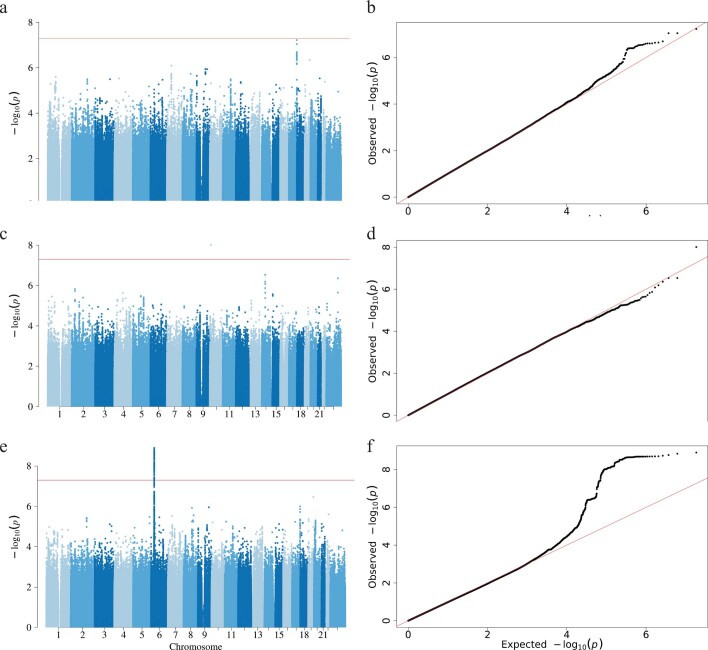
Testing effect of population stratification on HLA association signal in COV001 and COV002 participants. The Manhattan (**a**) and QQ plot (**b**) of the association tested using a linear-mixed model with RBD in ChAdOx1 nCoV-19 vaccinated individuals from COV001 and COV002 excluding the extended MHC region to determine whether the MHC signal may be contributing significantly to the increased genomic inflation factor in the complete association analysis. The Manhattan (**c**) and QQ plot (**d**) for the association analysis with quantitative N levels to test for spurious statistical inflation in the multi-ethnic cohort. The Manhattan (**e**) and QQ plot (**f**) for RBD including the first ten principal components of genetic variance as fixed effect covariates in the mixed model association analysis. The horizontal red lines in **a**, **c** and **e** represent the threshold of GWAS significance (*P* < 5 × 10^−8^) to account for the multiple tests performed.

These genetic association signals may be falsely observed as a result of two important factors. First, although every effort was made to normalize the antibody levels while acknowledging the different platforms for antibody measurement, the final distributions still deviated from normality, which could increase the risk of detecting a genetic association signal by chance (Extended Data Fig. 1). Therefore, we performed a further round of inverse normal transformation on the pooled RBD-specific antibody distributions (Extended Data Fig. 1f) to create a merged, normalized distribution (Extended Data Fig. 4a) and re-ran the association analysis for anti-RBD antibodies. The rs1130456 association was still present and was the most significant association (*P* = 4.7 × 10^−9^; Extended Data Fig. 4b–d). Second, given the diverse ancestry of individuals included, it is possible that the association could be a result of confounding due to population structure. The genomic inflation factor (λ) from our primary RBD association analysis was 1.023. When the extended MHC region was excluded, λ was 1.007, suggesting that much of the observed inflation was a result of the large number of variants associated with MHC (Extended Data Fig. 5a for Manhattan and Extended Data Fig. 5b for QQ). Furthermore, given the low levels of natural exposure to SARS-CoV-2 in our population at the time of sampling early in the pandemic, we used our data for anti-N IgG concentrations to again test for excessive inflation, using N as a negative control. No associations of genome-wide significance were observed, and λ was estimated at 1.017 (Extended Data Fig. 5c,d). To further explore the effect of population structure, we re-ran our association analyses for RBD, including the first ten genetic principal components (PCs) (derived from the entire genotyped dataset) as additional fixed-effect covariates using our mixed-model approach (Extended Data Fig. 5e,f). λ using this approach was 0.985, suggesting a degree of overfitting, but, again, the same variant (rs1130456) remained most significantly associated with RBD-specific antibodies, albeit with a marginally attenuated *P* value (1.3 × 10^−9^), as to be expected when including multiple additional covariates in the model.

### HLA imputation and fine-mapping of associated variants

We proceeded to test for evidence of association with spike-specific and RBD-specific IgG antibodies at the level of HLA gene and protein variation. Imputation (Methods) identified 640 HLA alleles and 4,513 amino acid (AA) changes (of which 81 alleles and 3,027 AA substitutions were present in our dataset at a minor allele frequency of ≥0.05). To undertake fine-mapping, we identified 1,023 individuals with identity-by-descent (IBD) values of 0.185 or less, and those of self-reported, and principal component analysis (PCA)-derived European ancestry (using PC1 and PC2 cutoffs as shown in Extended Data Fig. 2, inset). Of all HLA and AA alleles tested for association with spike and RBD antibody levels, the HLA allele with the most significant association was HLA-DQB1*06 with anti-RBD antibodies (*P* = 3.2 × 10^−9^, beta = 0.27 and s.e. = 0.04; Supplementary Table 1). An AA variant had a *P* value identical to that of HLA-DQB1*06 (3.2 × 10^−9^) but the exact inverse beta coefficient (−0.27). This AA variant (DQB1-125A/S) denotes the presence of either an alanine or a serine at position 125 of the HLA-DQB1 protein according to international ImMunoGeneTics (IMGT) project coordinates. HLA-DQB1*06 has a glycine at position 125, whereas other alleles common in our genotyped population possess either alanine (HLA-DQB1*02 and *04 alleles) or serine (HLA-DQB1*05). Thus, this AA variant is synonymous with the presence of HLA-DQB1*06 in our dataset. The index-associated variant from the primary analysis (rs1130456) was equally associated with the anti-RBD titers in this analysis (beta = 0.27 and s.e. = 0.04). Other variants imputed using the specific HLA imputation algorithm were identified as being marginally more significantly associated than rs1130456, with the new lead being rs9273817 (*P* = 2.4 × 10^−9^, beta = 0.27 and s.e. = 0.04).

**Fig. 3:**
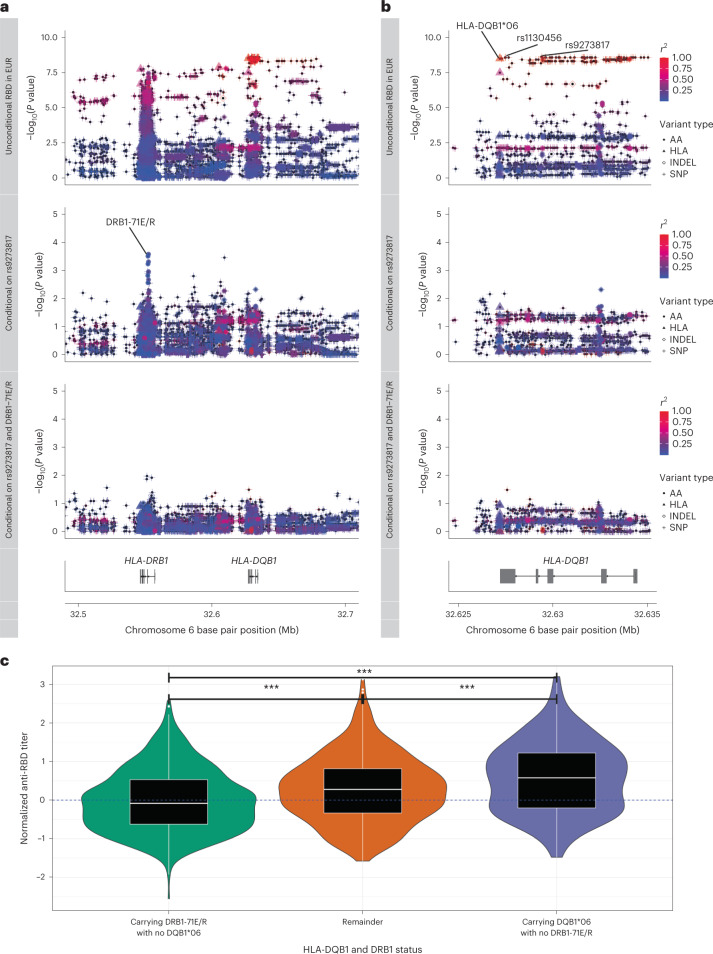
Fine-mapping the likely causal variants associated with day 28 post-prime anti-RBD antibody levels (normalized within immunoassay performed at MSD and PPD laboratories) in COV001 and COV002. **a**,**b**, Stepwise conditional analyses using linear regression were performed in 1,023 individuals restricted by self-reported White ethnicity and PCA axes and with IBD values less than or equal to 0.185. The primary unconditional association analysis across the class II MHC region (**a**) and HLA-DQB1 locus (**b**) is shown in the top rows, with points shaped by variant type (AA, HLA allele (HLA), insertion–deletion (INDEL) or SNP) and colored by linkage disequilibrium (*r*^2^) with the index variant (rs9273817). The key variants of interest (rs9273817, rs1130456 and HLA-DQB1*06) are highlighted. The middle and bottom rows of **a** and **b** represent the same points after adjustment for rs9273817 (middle row) and also DRB1-71E/R (bottom row) using the same shape and color definitions as the first row. **c**, 1,076 individuals from COV001/COV002 grouped by carriage of either DQB1*06 or DRB1-71E/R in absence of the other demonstrate the most significant differences between groups tested using the two-sided Student’s *t*-test as shown by violin plots overlain by box plots. The box plot center line represents the median; the box limits represent the upper and lower quartiles; and the whiskers are the 1.5× IQR. ***P < 0.001. EUR, European.

To further understand the relationship between the top associated variants, we performed stepwise forward regression analysis incorporating the full set of SNP, AA and HLA allele variants from HLA imputation in the set of individuals restricted by IBD, PCs and self-reported ethnicity (Fig. 3a,b). Adjusting for the new top SNP (rs9273817) in the first round of conditional analysis, there was a complete abolition of the signals for both rs1130456 (*P* = 0.65) and HLA-DQB1*06 (*P* = 0.65), supporting the likelihood that these variants are all tagging the causal variant, most plausibly HLA-DQB1*06 (Fig. 3a,b, middle rows). A second, likely independent, signal of association was observed in HLA-DRB1, with the index variant being the presence of a glutamate or an arginine at position 71 (according to IMGT) of HLA-DRB1 (DRB1-71E/R, *P*_conditional_ = 2.7 × 10^−4^, beta = −0.14 and s.e. = 0.04; Fig. 3a, middle row). After conditioning on this variant, no further independent signals with a *P* < 1 × 10^−2^ were observed in the class II MHC region. Before proceeding with further fine-mapping, we confirmed that both the HLA-DQB1*06 and DRB1-71E/R associations were robust to statistical inflation by performing a Monte Carlo exact test with 10^8^ permutations. The likelihood of both associations occurring by chance was still less than the number of permutations (that is, *P* < 2 × 10^−8^), limited only by computational time for testing. Furthermore, we tested for evidence of both the HLA-DQB1*06 and DRB1-71E/R associations in the different population strata (including 928 European versus 148 non-European) individuals and observed evidence of the same trend of association in both groups, supporting further that this association is unlikely to be spurious due to population structure (Supplementary Fig. 2).

Given our findings from the stepwise conditional analysis, we next sought evidence to further substantiate the effect being driven by the DQ locus and the relationship of the two independent associations to each other. First, HLA-DQB1*06 is frequently inherited as part of a common haplotype with HLA-DQA1*01 (with DQA1*01:02 being associated with anti-RBD antibody levels in our discovery dataset with *P* = 1.3 × 10^−8^, beta = 0.28 and s.e. = 0.05) and HLA-DRB1*15 (DRB1*15:01 *P* = 1.8 × 10^−8^, beta = 0.31 and s.e. = 0.05). These three alleles were most significantly associated with anti-spike antibody levels (*P* = 2.0 × 10^−7^, 7.8 × 10^−8^ and 4.6 × 10^−8^, respectively; Supplementary Table 2). We assessed the accuracy of HLA allele imputation by analyzing 60 individuals from the genotyped COV001 and COV002 vaccinees (120 alleles) who also underwent classical HLA typing (Supplementary Notes). The agreement between two-digit calls was 99.2% and 96.7% for HLA-DQB1 and HLA-DRB1 loci, respectively (Supplementary Table 3). The accuracy of calling the specific common HLA-DQB1*06:02 group of alleles to four-digit resolution was 97.0% and 100% for HLA-DRB1*15:01 alleles (Supplementary Table 4).

**Fig. 4:**
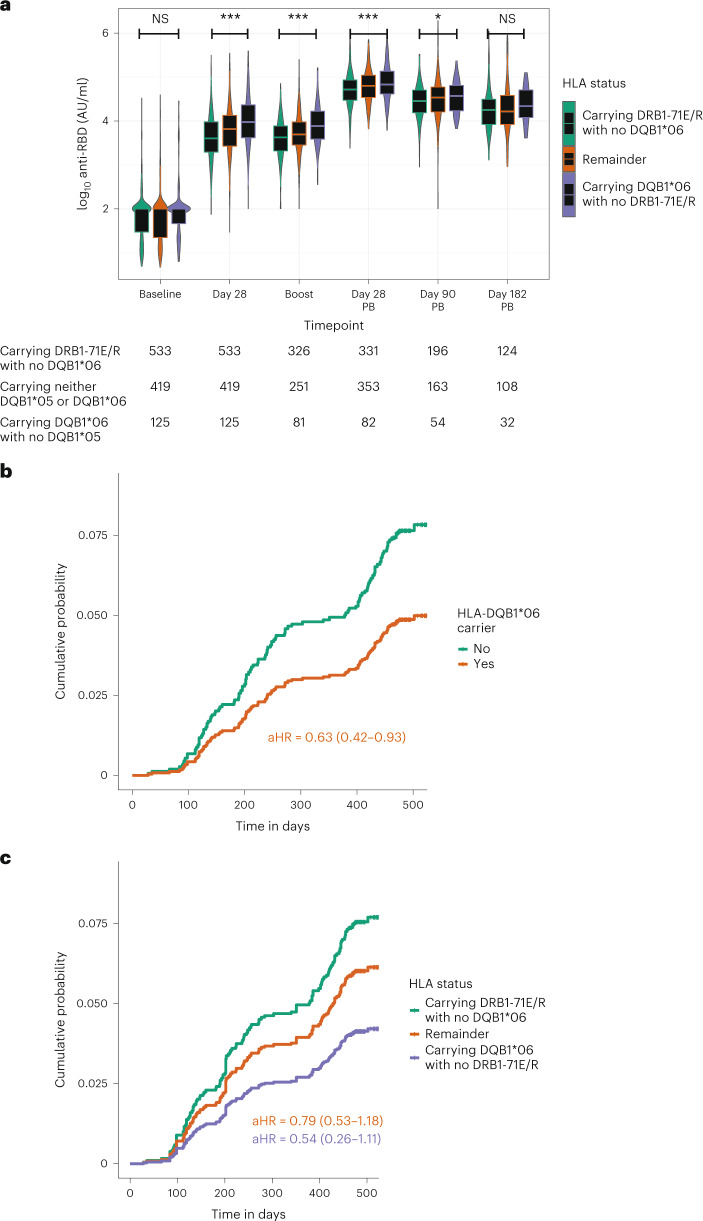
The effect of HLA-DQB1*06 on anti-RBD antibody accounting for DRB1-71E/R persists over time and influences risk of breakthrough infection in COV001 and COV002 in genotyped vaccine recipients. **a**, Where PPD-measured anti-RBD antibody levels were available in COV001 and COV002, the differences in vaccine responses by HLA type persisted over time. Differences were tested between the categories ‘Carrying DRB1-71E/R with no DQB1*06’ and ‘Carrying DQB1*06 with no DRB1-71E/R’ using the two-tailed Student’s *t*-test. Times of sampling are after either first or second (post-boost (PB)) vaccine doses. **b**,**c**, Adjusted Cox regression curves with risk of breakthrough infection over time in 1,069 individuals stratified by carriage of HLA-DQB1*06 (**b**) and HLA-DQB1*06 (**c**) alleles, accounting for DRB1-71E/R status in COV001 and COV002 vaccine recipients adjusted for age, sex, reported ethnicity, healthcare worker status, BMI and chronic disease status and including sample weighting for dose and interval between prime and boost vaccination. Included individuals had breakthrough infection at least 22 days after first vaccination. Box plot center line indicates median; box limits indicate upper and lower quartiles; and whiskers indicate 1.5× IQR. **P < 0.01 and *P < 0.05. aHR, adjusted hazard ratio; AU, arbitrary units; NS, not significant.

To confirm that the HLA-DQB1*06 allele was the most likely primary gene locus associated with the antibody response, we used Bayesian information criterion (BIC) modeling with phased data. We found that variation in RBD antibody levels was best described using HLA-DQ alleles (HLA-DQA1*01/HLA-DQB1*06, BIC 2,715.2) rather than HLA-DRB1 (HLA-DRB1*15, BIC 2,719.1), supporting that the primary association was likely linked to HLA-DQ, rather than HLA-DR, variation (Supplementary Fig. 3). We next investigated whether there was evidence of interaction between the HLA-DQB1*06 and independent DRB1-71E/R associations. Using a likelihood ratio test (LRT) comparing the linear and interaction terms, we found no evidence of a complex inter-dependence between these two variants (*P* = 0.44; Supplementary Fig. 4). We, therefore, compared models describing variation in a simple linear additive model (that is, normalized anti-RBD antibody levels ~ HLA-DQB1*06 + DRB1-71E/R) compared to a model where we compared individuals grouped into the presence of one variant in the absence of the other (Fig. 3c), and we found that the latter was more parsimonious after adjusting for age, sex, five PCs and anti-RBD antibody measurement assay (BIC 2,965.42 versus 2,689.65, respectively). Thus, using this combined description of variation, we next tested for association of HLA-DQB1*06 with increased anti-RBD antibody levels accounting for DRB1-71E/R over the time course of the ChAdOx1 nCoV-19 trial. We found significant differences between the opposing DQB1*06 and DRB1-71E/R carrier groups seen at day of second dose (*P* = 2.7 × 10^−7^ using the Student’s *t*-test), at day 28 after second dose (*P* = 2.6 × 10^−7^) and at day 90 after second dose (*P* = 0.01) (Fig. 4a). No significant difference was observed at day 182 after second dose. A summary of the baseline demographics of individuals stratified by HLA allele group is provided in Supplementary Table 5.

### Replication of genetic association signals with COVID-19 vaccination antibody responses

**Extended Data Fig. 6:**
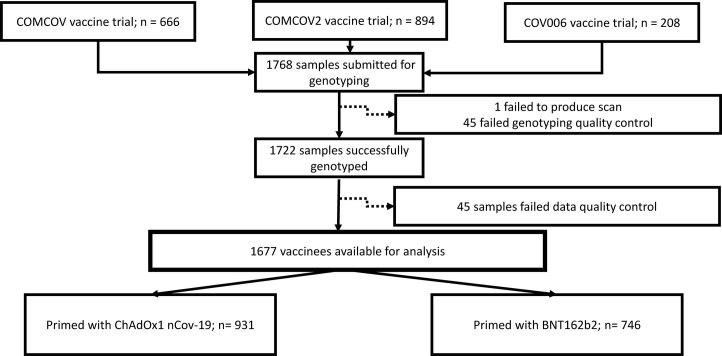
Flow diagram of participants from COMCOV, COMCOV2 and COV006 selected for replication analysis.

To provide further evidence that the observed genetic associations were robust and not restricted to the COV001 and COV002 cohorts, we aimed to test for replication of the associations in a series of independently recruited cohorts (Extended Data Fig. 6). Three trials were coordinated after COV001 and COV002 to address questions regarding safety and immunogenicity of heterologous dosing of COVID-19 vaccines (COMCOV and COMCOV2) and to assess immunogenicity and safety in children (COV006). DNA from 1,722 individuals (638 COMCOV, 876 COMCOV2 and 208 COV006) were genotyped on the same Affymetrix AxiomTM HGCoV2 1 array with identical quality control and imputation pipelines applied as to the COV001 and COV002 cohorts. After quality control, data from 1,677 individuals (627 COMCOV, 847 COMCOV2 and 203 COV006) were available (Supplementary Table 6). These replication cohorts differed considerably from the ChAdOx1 nCoV-19 trials with respect to age, sex proportions and timing and nature of vaccination regimens, and only data for spike antibody levels (and not RBD) were available. Moreover, COMCOV was enriched for non-White reported ethnicities. Nevertheless, when using the same HLA-DQB1*06 and DRB1-71E/R classifications, we observed statistical evidence of association with anti-spike antibody levels measured at the time of second vaccination in the same direction as observed for COV001 and COV002 when looking at those individuals receiving both ChAdOx1 nCoV-19 or BNT162b2 as their first vaccine. Both unadjusted and adjusted models were used to compare the groups as follows.

When including all individuals irrespective of the first vaccine received (all individuals genotyped from COMCOV, COMCOV2 and COV006 received either ChAdOx1 nCoV-19 or BNT162b2 as their first vaccine), there was a significant difference in anti-spike antibody levels measured on the day of second vaccine (median of 67 days with a range of 28–184 days after the first vaccine) when comparing the group ‘Carrying DRB1-71E/R with no DQB1*06’ against both those ‘Carrying DQB1*06 with no DRB1-71E/R’ (*P*_*t*_ = 5.4 × 10^−3^; *P* value derived from the *t*-test) and the ‘Remainder’ of individuals (*P*_*t*_ = 0.02; Supplementary Fig. 5a). Significant differences in anti-spike antibody levels were also observed for those individuals given a first dose of BNT162b2 comparing the groups ‘Carrying DRB1-71E/R with no DQB1*06’ and those ‘Carrying DQB1*06 with no DRB1-71E/R’ (*P*_*t*_ = 0.01) and the ‘Remainder’ of individuals (*P*_*t*_ = 0.04; Supplementary Fig. 5b) and for ChAdOx1 nCoV-19 with significance observed only for the comparison between ‘Carrying DRB1-71E/R with no DQB1*06’ and ‘Carrying DQB1*06 with no DRB1-71E/R’ groups (*P*_*t*_ = 0.04; Supplementary Fig. 5c). Using a linear model adjusting for age, sex, interval between vaccination and sampling, first vaccine, study and self-reported ethnicity, there was a significant difference in spike antibody levels observed for all individuals when testing the effect of HLA-DQB1*06 accounting for DRB1-71E/R (*P*_*LRT*_ = 5.2 × 10^−3^ using the LRT to compare with the null) and for those individuals primed with BNT162b2 (*P*_*LRT*_ = 0.02), with a trend toward significance for individuals primed with ChAdOx1 nCoV-19 (*P*_LRT_ = 0.1). Similarly, we observed a significant effect in the same direction when performing the same analysis comparing individuals grouped by either HLA-DQB1*06 carriage alone (*P*_*LRT*_ = 0.04) or DRB1-71E/R carriage alone (*P*_*LRT*_ = 7.8 × 10^−4^). All together, we found further evidence that carriage of HLA-DQB1*06 and the presence of either a glutamate or an arginine at position 71 in the HLA-DRB1 protein is associated with differential antibody responses to spike across individuals, irrespective of the nature of first vaccine and across ages and both self-reported White and non-White ethnicities. Given the correlation in antibody levels against spike and RBD in our discovery set, it is likely that our observed genetic signal would also be observed for RBD, even though RBD was not measured directly in the replication cohorts.

### Testing for association of genetic variants on immune response over time and risk of breakthrough infection

**Extended Data Fig. 7:**
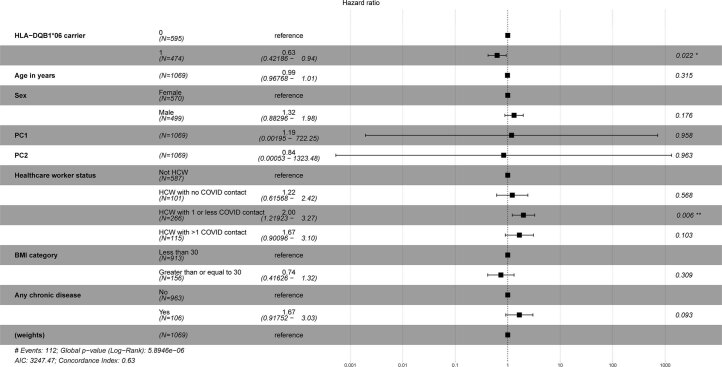
Effect estimates with 95% confidence intervals of covariates and HLA-DQB1*06 carriage in an adjusted Cox proportional hazards regression model predicting breakthrough infection in genotyped vaccine recipients from COV001 and COV002.

**Extended Data Fig. 8:**
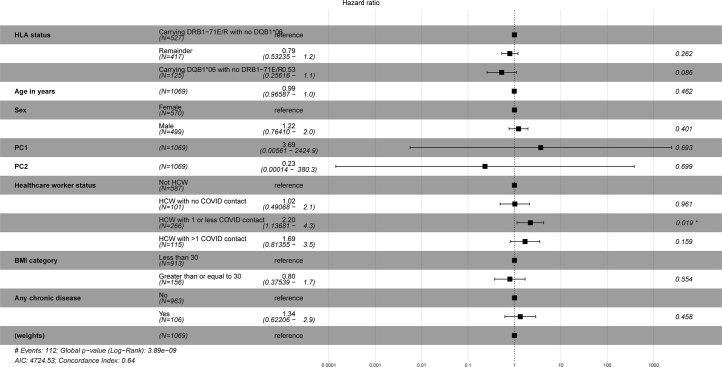
Effect estimates with 95% confidence intervals of covariates and carriage HLA-DQB1*06 accounting for DRB1-71E/R in an adjusted Cox proportional hazards regression model predicting breakthrough infection in genotyped vaccine recipients from COV001 and COV002.

**Extended Data Fig. 9:**
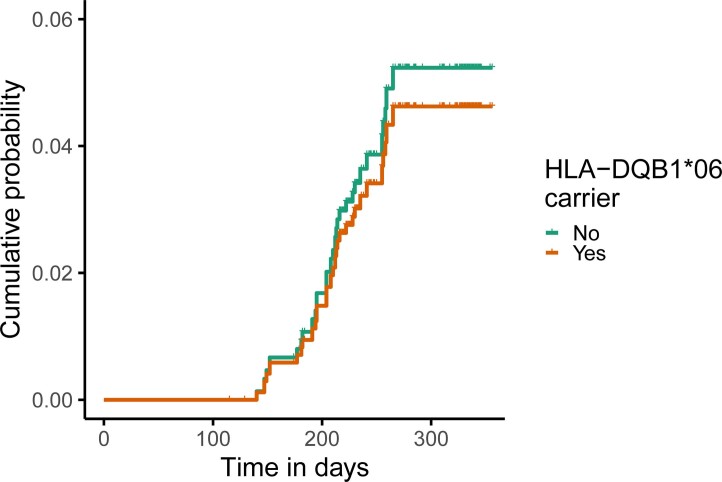
Risk of breakthrough by HLA-DQB1*06 carriage in the replication cohort individuals. Adjusted Cox regression curves with risk of breakthrough infection in 401 individuals less than 55 years of age and self-reporting White ethnicity over time stratified by carriage of HLA-DQB1*06 alleles in merged COMCOV/COMCOV2/COV006 vaccine recipients. Curves are adjusted for age, sex, first vaccine type (BNT162b2 or ChAdOx1 nCoV-19) and second vaccine (viral vector (ChAdOx1 nCoV-19), mRNA (BNT162b2 or mRNA-1273) or nanoparticle (NVXCoV2373)) received as covariates with reweighting performed using interval between first and second vaccines in days.

Given the observed association between HLA variants with variable immunogenicity, we next investigated whether there was a relationship with risk of breakthrough infection. At a median of 494 days (interquartile range (IQR), 479–535) of follow-up from the first dose of vaccine in participants from the trials used in the discovery analysis (COV001 and COV002), 112 episodes of breakthrough infection (individuals with a SARS-CoV-2 nucleic acid amplification test (NAAT)-positive swab at least 22 days after a first dose of vaccine, with the prevalent ancestral virus and Alpha variants) had been recorded in genotyped individuals who had received the ChAdOx1 nCoV-19 vaccine (Table 1 and Supplementary Table 7). We found that HLA-DQB1*06 was present in 33.9% of individuals experiencing breakthrough infection compared to 45.6% of individuals who did not have breakthrough infection (chi-squared *P* = 0.02). We subsequently found that individuals carrying HLA-DQB1*06 alleles were less likely to experience breakthrough infection over time compared to individuals who did not carry HLA-DQB1*06 after adjustment for age, sex, the first two genetic PCs (representing ethnicity), healthcare worker status, body mass index (BMI) and chronic disease status (adjusted hazard ratio (HR) = 0.63, 0.42–0.93, *P* = 0.02; Fig. 4b and Extended Data Fig. 7). We performed sample reweighting for dose and interval between first and second dose of vaccination (using inverse probability weighting) to ensure that our analyses were as consistent with prior correlates analyses as possible^13^. This significance persisted even after adjusting for whether individuals were likely to have been naturally exposed to SARS-CoV2 (determined using N measurements) and based on whether they were related to each other (IBD < 0.185) or not (*P* = 0.02). A similar effect was observed when describing individuals using our overall HLA status definition (that is, carrying HLA-DQB1*06 alleles accounting for DRB1-71E/R), although significance was lost (adjusted HR for the group ‘Carrying DRB1*06 with no DRB1-71E/R’ was 0.54, 0.26–1.1, *P* = 0.09; Fig. 4c and Extended Data Fig. 8). The lower frequency of HLA-DQB1*06 in individuals experiencing breakthrough infection was observed both in the 41 individuals meeting the definition of primary symptomatic breakthrough infection (31.7% carrying HLA-DQB1*06 among those with the primary definition of breakthrough infection) and in the 66 individuals who were asymptomatic (28.8%) but not in the nine individuals who did not meet the primary definition of symptomatic breakthrough (66.7%) (Supplementary Table 7). To further substantiate our finding, we explored whether we could find any evidence of an equivalent effect in the tested replication cohorts, acknowledging that the cohorts differed from ChAdOx1 nCoV-19 not only in regard to age, ethnicity, comorbidities and the predominant circulating SARS-CoV-2 variant (Alpha and Delta) but also because breakthrough infection was defined through self-report rather than active surveillance. In the subset of individuals of self-reported White ethnicity and less than or equal to 55 years of age (thus enriching for individuals more representative of the COV001 and COV002 trial, *n* = 401), there were 29 individuals experiencing breakthrough and 372 individuals with no breakthrough reported after a median of 280 days of follow-up (IQR, 244–332). HLA-DQB1*06 was observed in 37.9% of individuals experiencing breakthrough infection and 40.3% in individuals with no breakthrough infection. After adjustment for age, sex, first vaccine received (ChAdOx1 nCoV-19 or BNT162b2) and booster received (viral vector (ChAdOx1 nCoV-19), mRNA (BNT162b2 or mRNA-1273) or nanoparticle (NVXCoV2373)) with reweighting calculated based on days between first and second doses of vaccine, carriage of HLA-DQB1*06 had an adjusted HR of 0.87 (0.41–1.80, *P* = 0.73) of risk of breakthrough infection (Extended Data Fig. 9).

### Structural insights into HLA spike peptide binding

**Fig. 5:**
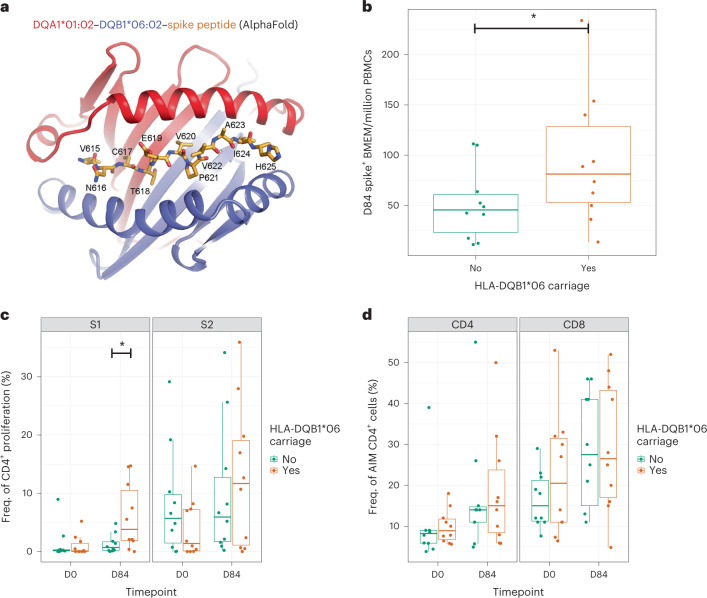
The clinical implications and mechanisms of the HLA associations with differential spike/RBD antibody levels. **a**, AlphaFold-based model of HLA-DQA1:01:02–HLA-DQB1:06:02–spike peptide. The peptide is shown in orange. Residue numbering corresponds to UniProt ID P0DTC2. Memory B cell (**b**), CD4^+^ T cell proliferation (**c**) and AIM CD4^+^ T cell (**d**) responses using biologically independent samples from 20 individuals from COV001 and COV002 stratified by carriage of HLA-DQB1*06 allele carriage sampled at days 0 and 84 after first vaccine, with significant differences tested for using a one-sided Wilcoxon rank test. Statistical differences were seen between HLA carriage groups for the memory B cell responses (**b**, *P* = 0.05) and S1 proliferation response (**c**, *P* = 0.01) at day 84. Box plot center line indicates median; box limits indicate upper and lower quartiles; and whiskers indicate 1.5× IQR. *P < 0.05.

**Extended Data Fig. 10:**
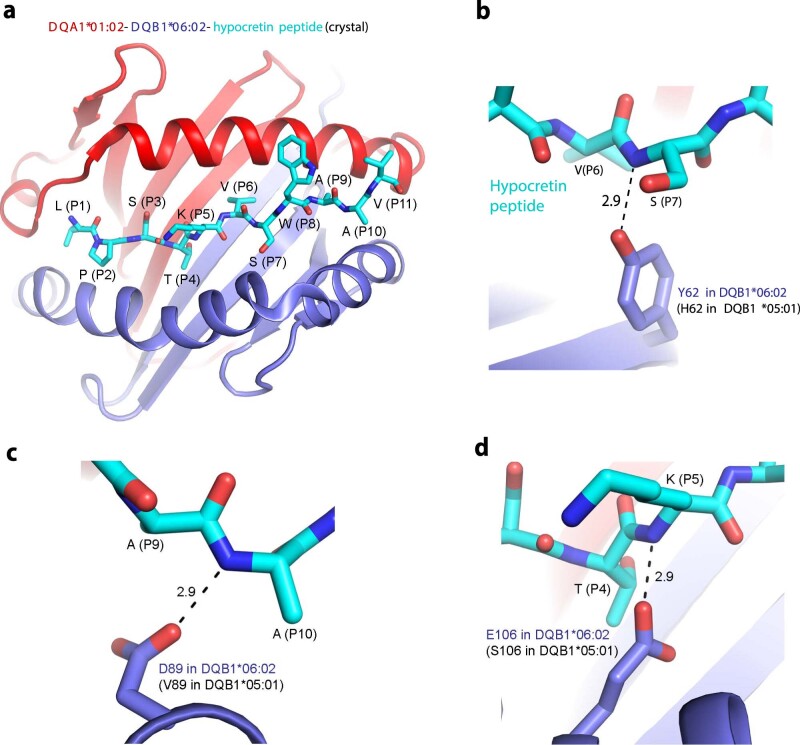
Crystal structure and HLA-DQA1:01:02–HLA-DQB1:06:02–peptide interactions. **a**) the crystal structure of HLA-DQA1:01:02–HLA-DQB1:06:02–hypocretin peptide (Protein Data Bank ID 1UVQ) coloured in red, blue, and cyan, respectively. Side chains of the peptide are shown as sticks and numbered. Carbon atoms of the peptide are coloured in cyan (oxygen, red; nitrogen, blue). **b**)**–d)**, hydrogen bonds between the hypocretin peptide and HLA-DQB1:06:02 are indicated with dashed lines with distances between selected atoms shown in Å. Numbering of HLA-DQB1:05:01 and HLA-DQB1:06:02 correspond to UniProt IDs Q5Y7F1 and Q5Y7D6, respectively.

Given the observed immunological and clinical impact of HLA-DQB1*06 on vaccine response and effectiveness, we next tested for structural evidence of binding of spike peptides by the associated HLA-DQB1*06 allele. We tested the hypothesis that HLA-DQB1*06:02 could bind peptides from SARS-CoV-2 spike more effectively than an alternative HLA-DQB1 allele that was both common in the population and linked with another HLA-DQA1*01 allele. Using the COV001/COV002 data, we identified HLA-DQB1*05:01 as an allele that would act as a suitable comparator (frequency in COV001/COV002 = 12%, beta for association with 28-day RBD levels = −0.14, s.e. = 0.05 and *P* = 0.01, acknowledged to commonly pair with HLA-DQA1*01:01 (ref. ^28^)). Other HLA alleles that were common in our merged COV001/COV002 dataset were less suitable for this analysis. DQB1*03, for example, (frequency, 34%) pairs more commonly with DQA1*03 or *05 alleles, whereas DQB1*02 (23%) pairs with DQA1*02 or DQA1*05. The only available crystal structure for DQB1*06 alleles is the HLA-DQA1*01:02–HLA-DQB1*06:02 in complex with a hypocretin peptide (LPSTKVSWAAV)^29^. The key sidechains of Ser (position (P) 3), Thr (P4) and Val (P6) of the peptide are buried in the center of the groove formed by two HLA molecules (Extended Data Fig. 10a). Thus, we searched for a hypocretin-like peptide motif (Ser/Cys) ThrXVal in spike protein (where X is any amino acid with its sidechain pointing away from the groove; Ser and Cys differ in one atom only). Spike residues Val615-NCTEVPVAI-His625 could be aligned with a hypocretin peptide and, thus, enabled us to model a complex of HLA-DQA1*01:02–HLA-DQB1*06:02 bound to the spike peptide using AlphaFold^30^ (Fig. 5a). Both the AlphaFold-based model and the crystal structure support DQB1*06:02 interacting differently with any peptide compared to DQB1*05:01. DQB1*05:01 differs from DQB1*06:02 by at least three key residues forming hydrogen bonds with the bound hypocretin peptide (Extended Data Fig. 10b–d), making analogous DQB1*05:01–peptide interactions impossible. Our analysis identifies specific residues of DQB1*05:01 and DQB1*06:02 responsible for different peptide recognition and subsequent recognition by T cell receptors.

### Immunological implications of the observed MHC associations

To further support these observations, we used peripheral blood mononuclear cells (PBMCs) available from a small number of participants from COV001 and COV002 to compare anti-spike-specific memory B cell responses at day 84 after the first vaccine in ten individuals carrying HLA-DQB1*06 and ten individuals not carrying HLA-DQB1*06. We observed an increase in anti-spike memory B cell responses in the individuals carrying HLA-DQB1*06 (*P* = 0.05 using a one-tailed Wilcoxon rank test) at day 84 that was not apparent at day 0 (Fig. 5b). We then searched for similar signals of association in the intermediate components of the MHC–T–B–antibody axis. We observed a difference in overall CD4 proliferation in response to stimulation with S1 (that includes the RBD domain and the putative Val615-NCTEVPVAI-His625 peptide, *P* = 0.01; Fig. 5c) but not against S2 (cleaved away before residue 686). We did not observe an equivalent signal with antigen-specific T cell activation (using the activation-induced marker (AIM) assay; Fig. 5d).

## Discussion

Our findings show that individuals carrying HLA-DQB1*06 alleles have higher antibody responses against SARS-CoV-2 spike protein and the RBD after vaccination with both ChAdOx1 nCoV-19 and BNT162b2 vaccines than individuals not carrying this allele. HLA-DQB1*06 is also associated with a reduced risk of breakthrough infection based on PCR positivity after a median 494 days of follow-up after receiving their first dose of vaccine. To our knowledge, this is the first report of an HLA association with antibody responses after immunization with SARS-COV-2 vaccines and of a genetic association with risk of SARS-CoV-2 breakthrough infection^15–18,22–24,31^. We further provide a working mechanistic hypothesis for the primary HLA-DQB1*06 association of potentially distinct peptide binding that may lead to improved CD4^+^ T cell proliferation and memory B cell activation. Our study design comprised an infection-naive and vaccine-naive population in a clinical trial setting with appropriate blinding, detailed immune phenotyping and patient follow-up at defined timepoints, and we further substantiated our findings in a large replication dataset with preliminary follow-up functional experiments.

The global evidence of breakthrough infections after vaccination, of changes in immune correlates of protection over time and of new SARS-CoV-2 variants highlight the importance of subsequent dosing of vaccination and understanding how this can be optimally deployed^9,13,32^. Our study demonstrates that there is a heritable component to observed inter-individual variation in antibody response at day 28 after first dose but also throughout time after vaccination, across vaccinee age and type of first vaccine. Given that these effects do persist over time, with some change in effect size, and have clinical relevance in terms of risk of breakthrough infection, the observed HLA associations raise the potential utility of prioritizing at-risk populations based, for example, on HLA-DQB1*06 allele frequencies, among whom more intensive booster vaccination may be warranted. Variable HLA-DQB1*06 allele frequencies are reported across diverse populations^33–36^ but there is not yet robust epidemiological evidence of the extent of breakthrough infections in such populations, and this would require further investigation before implementation of such an intervention. The observed reduction in effect size on log_10_-transformed RBD-specific antibody levels in the ChAdOx1 nCoV-19 vaccinees over time from 0.38 at day 28, to 0.32 at boost, to 0.17 at day 28 after boost and to 0.12 at day 90 may represent a true reduction in effect of genetic variation over time or could also be a result of the limit of detection and dynamic range of the antibody assay. Further re-analyses using recalibrated assay detection systems would be necessary to resolve this issue.

Although we have provided preliminary evidence for our mechanistic hypothesis for HLA-DQB1*06, further studies to understand the structure–function relationships based on the specific allele/peptide predictions and T cell biology will be required. Previous genetic studies for non-SARS-CoV-2 vaccines implicating the MHC suggest both HLA peptide binding^23,37^ and non-HLA effects relating, for example, to differential gene expression or complement activation^24^, as potential underlying mechanisms for observed genetic associations. For SARS-CoV-2 vaccine response, consideration of HLA genotype has been advocated in vaccine design based on predicted antigens presented to T cells across different ethnic groups to maximize efficacy based on T cell immunity, with potential utility as a booster agent to strengthen immune responses^38^.

Limitations of the study include the need for further replication of the genetic association in other studies and populations and the representativeness of the trial population to the wider UK and global population. We propose that there is an urgent need to investigate these associations further in diverse ethnic groups and individuals of varying comorbidity to maximize insights and potential utility of the observed associations. There is also a requirement for mechanistic studies to further understand the functional basis of the association and the relationship with specific SARS-CoV-2 variants. A further limitation is the extent to which fine-mapping the association to specific variants and modulated genes was possible, reflecting the high level of sequence and structural polymorphism, sequence homologies and complex linkage disequilibrium in the MHC^39^. Only two antibody responses were analyzed, with a greater antibody repertoire and T cell immune response assays and other aspects of cell-mediated immunity important to include in future studies.

We propose that, to inform vaccine design and implementation against COVID-19 and other vaccine-preventable diseases with products either established or in development, an understanding of the impact of human genetics should be prioritized to deliver translational outputs for the long-term benefit of populations worldwide.

## Methods

### Study design and participants

#### Discovery cohort

The participants were enrolled in phase 1/2 (COV001) or phase 2/3 (COV002) randomized single-blind ChAdOx1 nCoV-19 (AZD1222) vaccine multi-center efficacy trials conducted across multiple sites within the UK^4,40^ (NCT04324606 and NCT04400838). In brief, after written informed consent, adults aged 18 years and older were randomly assigned to receive either intramuscular ChAdOx1 nCoV-19 (AZD1222) or a control vaccine (MenACWY) to assess the safety and efficacy of the ChAdOx1 nCoV-19 vaccine against SARS-CoV-2 (refs. ^4,40^). All individuals included in the analyses agreed to being included in genetic studies as part of the vaccine trial consent, with the opportunity to opt out. The trials were conducted according to the principles of Good Clinical Practice and approved by the South Central Berkshire Research Ethics Committee (20/SC/0145 and 20/SC/0179) and the UK regulatory agency (the Medicines and Healthcare products Regulatory Agency). Individuals with humoral immune responses measured after vaccination were selected for this genotyping study. To maintain blinding of investigators to vaccine allocation—before vaccine trial reporting—participants who received the control vaccine were also genotyped, at a ratio of 1:10.

##### Breakthrough infections

Participants received weekly reminders to report any primary symptoms of COVID-19 (cough, shortness of breath, fever, anosmia or ageusia), and, if symptomatic, a SARS-CoV-2 NAAT was performed on a nose and throat swab. Participants were also asked to return a weekly nose and throat swab for NAAT for the duration of the study. A breakthrough infection was defined as SARS-CoV-2 NAAT-positive swab at least 22 days after a first dose of vaccine. If participants were NAAT-positive but had symptoms other than the five primary COVID-19 described above, they were categorized as non-primary symptomatic cases but still included in the final breakthrough analyses reported herein.

#### Replication cohort

The replication cohort comprised participants from three COVID-19 vaccine trials conducted across several sites within the UK^41–43^. Two of these trials (COMCOV and COMCOV2) were in adults aged 50 years and older, randomized to receive homologous or heterologous two-dose schedules of intramuscular ChAdOx1 nCoV-19, mRNA vaccines (BNT162b2 or mRNA-1273) or a nanoparticle vaccine (NVX-CoV2373)^42,43^. The other trial (COV006) was in children aged 6–17 years who were randomized to receive either intramuscular ChAdOx1 nCoV-19 or a control vaccine (capsular group B meningococcal vaccine, 4CMenB)^41^. All participants included in this manuscript consented (or their parents/guardians) to being included in genetic studies as part of the vaccine trial consent, with the opportunity to opt out in the consent form.

##### Breakthrough infections

Adult participants in the replication cohort self-reported breakthrough COVID-19 (no active COVID-19 testing as part of study) and associated symptoms. Parents/guardians reported PCR-confirmed or lateral flow assay-confirmed SARS-CoV-2 infections for the childhood participants. We defined a breakthrough infection for this cohort as a self-reported case of COVID-19 at least 22 days after a first dose of vaccine.

### Antibody concentrations

#### Discovery cohort

Blood samples for serological testing were taken at baseline, day 28 after the first dose of vaccine, before the second vaccine and then at days 28, 90 and 182 after the second dose of vaccine. Day 28 post-first-vaccine responses were available on all participants, and the variance of response across the cohort was substantial, and together this influenced the choice for the discovery genome-wide association study (GWAS). Humoral immune responses were assessed using Meso Scale Discovery (MSD) multiplexed immunoassay against SARS-CoV-2 spike and RBD as well as the N proteins—in the entire phase 1/2 UK cohort (*n* = 585) and 15% of the phase 2/3 UK cohort (*n* = 637). Sample selection from the phase 2/3 trial was based on samples processed for the initial application for emergency use, which required 15% of samples included in the efficacy trial to be processed on validated assays. In addition, serological testing was also performed on samples from NAAT-positive individuals—missing data were assumed to be missing at random.

Anti-spike, RBD and N IgG levels were measured by a multiplex immunoassay using the MSD platforms at two laboratories; the phase 1/2 samples were performed at MSD, and the phase 2/3 samples were performed at PPD Laboratories. Ninety-seven samples were assessed by both laboratories to evaluate inter-assay agreement.

#### Replication cohort

The replication cohort had blood samples for serological testing taken at baseline participation in the study, which was 28–84 days after the first dose of vaccine and on the day of receiving the second dose of vaccine. Adult participants in the replication cohort had their SARS-CoV-2 anti-spike IgG levels measured by enzyme-linked immunosorbent assay (ELISA) at Nexelis (Laval, Canada). In the childhood replication study, anti-spike IgG levels were measured using MSD at PPD Laboratories before receipt of any further vaccine dose.

### DNA extraction

DNA was extracted from either blood clots remaining after serum separation by centrifugation or whole blood collected in EDTA tubes. In brief, clots were dispersed by centrifugation through clot spin baskets (Qiagen, 158932). ATL buffer (Qiagen, 1014758) was added to the centrifuged clot and vortexed. Proteinase K (Qiagen, 19131) was added, vortexed thoroughly and incubated in a shaking incubator at 56 °C until the clot was completely lysed (overnight). Afterr lysis, AL buffer was added (Qiagen, 1038826) and vortexed thoroughly. Lysate or whole blood was then transferred to the QIAsymphony 2.0 and extracted using the QIAsymphony DSP DNA Midi Kit (Qiagen, 937255).

### Genotyping

Standardized DNA was sent to the National Institute for Health Research UK BioCentre and genotyped using their established pipelines on the Affymetrix AxiomTM HGCoV2 1 array. Raw CEL files were transferred back to Oxford for file conversion into build 38 using the Axiom Analysis Suite Best Practice Workflow. Individual samples and SNP variants were exported and underwent further quality control using PLINK^44^ (version 1.9).

Individuals were excluded if more than 3% of SNPs were classed as missing or if derived genetic sex did not match reported sex or if levels of heterozygosity lay more than three times the standard deviation from the mean of individuals stratified by self-reported ethnicity. IBD was calculated for all pairs of individuals, and individuals were excluded if they had estimates ≥0.9, excluding the individual with the highest SNP missingness rate from each pair preferentially. Hardy–Weinberg estimates were calculated for each SNP within individuals classified as founders (with IBD < 0.05), and SNPs were excluded if exhibiting extreme deviations from equilibrium (using a threshold of *P* < 1 × 10^−50^ calculated in PLINK 1.9).

PCs were calculated for all individuals both within the genotyped COVID-19 vaccine cohorts and merged with individuals from the 1000 Genomes Project derived from Great Britain (GBR), Han individuals from China (CHS) and African individuals from Kenya (LWK) and Nigeria (YRI). Before merging vaccinee data with 1000 Genomes individuals, SNPs were first lifted over from build 38 to build 37 coordinates using LiftOver (https://genome.sph.umich.edu/wiki/LiftOver). Plots were inspected to detect samples with extreme outlier values (>3 standard deviations from any expected cluster). A European cluster was defined by including only individuals falling within a defined cluster with GBR individuals and self-reporting as White ethnicity. Quality-controlled SNPs and individuals were taken forward for genotype imputation, which was undertaken on the Michigan Imputation Server using the TopMed reference panel using recommended settings^45^. Files were converted to MACH format using DosageConvertor (version 1.0.4, https://genome.sph.umich.edu/wiki/DosageConvertor).

HLA imputation was performed using the Multi-Ethnic HLA reference panel (version 1.0 2021) available on the Michigan Imputation Server^46^ using recommended settings. Phasing of multi-allelic HLA alleles was undertaken using PHASE (version 2.1.1)^47^. HLA typing was also performed using PCR sequence-specific primers (SSPs) at the Histogenetic laboratory, Oxford University NHS Foundation Trust.

### Structural analyses

The ternary HLA-DQA1*01:02–HLA-DQB1*06:02–spike peptide complex was modeled using AlphaFold^30^ available on Google Colaboratory server^48^. Structures were analyzed and displayed using the PyMOL Molecular Graphics System, version 2.3.2., from Schrödinger.

### Follow-up functional assays

Samples at baseline (day 0) and 28 days after boost (day 84) from 20 healthy adult volunteers participating in COV001 and COV002 who had received a two-dose ChAdOx1 nCoV-19 vaccine schedule of either two standard doses or one standard dose and one low dose were chosen for further functional work. Samples were evenly stratified by dose of second vaccine across individuals who either carried HLA-DQB1*06 (with no HLA-DQB1*05) or carried HLA-DQB1*05 (with no HLA-DQB1*06).

For the proliferation assay, cryopreserved PBMCs were washed twice with sterile DPBS and incubated with CellTrace Violet (Life Technologies), a free amine binding dye, at a final concentration of 2.5 μM in PBS for 10 minutes at room temperatuire, protected from light. To quench any dye remaining in solution, cells were incubated with FBS for 5 minutes at 4 °C. Cells were then resuspended in RPMI-1640 media supplemented with 1% L-glutamine, 1% penicillin–streptomycin and 10% human AB serum (Sigma-Aldrich), and 250,000 cells were seeded per condition in U-bottom 96-well tissue culture plates. Cells were stimulated with 15-mer peptides overlapping by ten AAs, comprising the length of the S1 or the S2 domain of the SARS-CoV-2 spike protein (ProImmune) at a final concentration of 1 μg ml^−1^. Two μg ml^−1^ of PHA served as a positive control, and cells incubated with 0.13% DMSO (Sigma-Aldrich) in cell media were used as a negative control. Cells were then incubated for 7 days at 37 °C, with 5% CO_2_ and 95% humidity, and the media was changed on day 4. At the end of the incubation period, cells were stained with anti-human CD3-FITC (OKT3, Invitrogen), CD4-APC (RPA-T4, Invitrogen), CD8-PE-Cyanine7 (OKT8, Invitrogen) and Live/Dead Fixable Near-IR Stain (Invitrogen) before acquisition on a five-laser LSRFortessa X-20 flow cytometer (BD Biosciences) using FACSDiva version 8.02 (BD Biosciences), and data were analyzed in FlowJo version 10.7. A hierarchical gating structure was applied, and data are presented as background subtracted.

For the T cell AIM assay, cryopreserved PBMCs from the same individuals were stimulated overnight (18 hours, 37 °C, 5% CO_2_) at 2 × 10^6^ cells per tube in rounded 5-ml polystyrene U-bottom tubes (Falcon). Samples were stimulated with anti-CD28 (CD28.2, 1 µg ml^−1^, Invitrogen) and anti-CD49d (9F10, 1 µg ml^−1^, Invitrogen) alongside either a SARS-CoV-2 S1/S2 peptide megapool (134 peptides, 2 µg ml^−1^ per peptide, ProImmune) or anti-CD3 (OKT3, Tonbo Biosciences) as a positive control.

Samples were stained with αCCR7-PE-Cy7 (G043H7, BioLegend) for 10 minutes at 37 °C before the addition of the following antibodies and incubated for 30 minutes at room temperature: αCD3-BV711 (UCHT1, BD Biosciences), αCD4-PerCP-Cy5.5 (RPA-T4, BioLegend), αCD8-BV650 (RPA-T8, BioLegend), αCD14-APC-Cy7 (HCD14, BioLegend), αCD19-APC-Cy7 (HIB19, BioLegend), Live/Dead Fixable Near IR Stain (Invitrogen), αCD137-PE-Cy5 (4B4-1, BioLegend), αOX40-APC (Ber-ACT, BioLegend), αCD69-BUV395 (FN50, BD Biosciences), αCCR6-PE (G034E3, BioLegend), αCXCR5-PE Dazzle 594 (J252D4, BioLegend), PD1-BV510 (EH 12.1, BD Biosciences), CXCR3-FITC (G025H7, BioLegend), CD45RA-AF700 (H1100, BioLegend) and CCR7-PE-Cy7 (G043H7, BioLegend). Samples were acquired using a BD LSRFortessa Cell Analyzer.

Memory B cells were differentiated into antibody-secreting plasma cells for the detection of IgG responses using enzyme-linked immunospot (ELISpot) according to the following steps. Cryopreserved PBMCs from the same individuals were isolated from heparinized whole blood and resuspended at a final concentration of 2 × 10^6^ cells per milliliter in Complete Medium (CM). CM was prepared by combining RPMI (450 ml, R-5886, Merck-Sigma), FBS-HI (50 ml, F9665, Merck-Sigma), penicillin–streptomycin (5 ml, P-4458, Merck-Sigma), L-glutamine (5 ml, G-7513, Merck-Sigma), NEAA (5 ml, 11140035, Life Technologies), sodium pyruvate (5 ml, 11360039, Life Technologies) and 2-mercaptoethanol (5,002 µl, 31350010, Life Technologies). Then, 10 ml of polyclonal stimulants antigen mix (CpG (tlrl-2006-5, InvivGen SAS) + SAC (507861-50, VWR International) + PWM (L-9370, Sigma-Aldrich)) was distributed in 1 × 96-well plate (650180, Greiner) at 100 µl per well. Then, 100 µl per well of cell suspension was added to each well (giving 2 × 10^5^ cells per well) and incubated at 37 °C/5% CO_2_/95% humidity for 5–6 days. Memory B cells were harvested from the 96-well plate by gentle re-suspension into a 30-ml universal container (201150, Greiner) and washed three times. The final pellet was re-suspended in 1 ml of wash buffer for cell counting and resuspended in CM at 2 × 10^6^ cells per milliliter. ELISpot was performed as previously described^49,50^.

### Statistical analyses

#### Discovery cohort

Antibody responses were log-transformed and density distribution plots inspected for overlap in density distributions between laboratories (MSD or PPD) and paired correlation between assays using results available from both laboratories performed on samples from the same individual. If the paired correlation was less than 0.7, or the density distributions did not overlap, traits were tested for association within assay type and then meta-analyzed, but if the paired correlation was greater than 0.7, and the density distributions overlapped, traits were quintile normalized (using the qqnorm() function in R) within assay platform groups and tested in a pooled analysis including assay type as a fixed-effect covariate. Samples taken at day 28 after first dose were used for the primary analysis.

A linear mixed model was used to maximize power and account for the diverse population structure and potential unrecognized close (cryptic) relatedness between study participants. Age, sex, antibody assay platform and N positivity were included as fixed-effect covariates for each association. Genotype and HLA-wide association analyses were performed using the ‘mlma’ function in GCTA (version 1.24.4)^51^ after generating a GRM using non-pruned genotyped SNPs. The GRM was modeled as a random effect covariate with age (in years), sex, antibody assay laboratory and likelihood of natural exposure (based on anti-N protein antibodies (anti-N) > 1,000) coded as a binary variable included as fixed-effect covariates for the primary GWAS.

Sensitivity analyses for the genetic components included a further round of association analyses incorporating the first ten PCs for all individuals calculated within-cohort and a further round of normalization on the within-assay normalized RBD distributions.

Comparisons between imputed and classically typed HLA alleles were undertaken at the four-digit (that is, two-field) level of resolution. If a classically typed available call at a single allele locus included several potential higher-resolution alleles (that is, a list of potential ambiguities), only the first available allele call (adhering to a Common and Well-Documented priority) was used for comparison. If types were available to six-digit or eight-digit resolution, the calls were reduced to four-digit resolution for comparison between call types. The classical types were treated as the ‘truth’ set. By comparing each individual allele in turn, it was possible to define calls of the imputed (or ‘test’) set that were:

- True positives (TPs)
- False positives (FPs); called by the test as that allele when it was, in fact, another allele according to the truth
- False negatives (FNs); called by the test as another allele when it was, in fact, this allele according to the truth
- True negatives (TNs)

Thus, at the level of an individual allele, various metrics could be calculated. Sensitivity was defined as:

$$TP/\left( {TP + FN} \right)$$

Specificity was defined as:

$$TN/\left( {TN + FP} \right)$$

Positive predictive value (PPV) was defined as:

$$TP/\left( {TP + FP} \right)$$

Negative predictive value (NPV) was defined as:

$$TN/\left( {TN + FN} \right)$$

Accuracy was defined as:

$$\left( {TP + TN} \right)/\left( {TP + FP + FN + TN} \right)$$

Concordance was calculated at the level of the locus. For every pair of chromosomes with data available in both truth and test sets, the number of identical allele calls between platforms was calculated and divided by the total number of alleles, equivalent to the PPV. Any individual with missing alleles on either or both chromosomes on either platform was excluded from these calculations.

Univariate *P* values were calculated using the Wilcoxon rank test for continuous variables and the chi-squared test for nominal variables. Permutation of Wilcoxon rank test of associating HLA alleles with RBD response was performed using the ‘perm’ package in R^52^. Adjusted Cox proportional hazards regression analyses for the breakthrough analyses were performed using the ‘survival’^53^ package in R, and plots were generated using ‘survminer’ and ‘ggplot2’. The breakthrough infection definition above for the discovery cohorts was used as the outcome in the proportional hazards model with individuals censored at time of breakthrough, date of withdrawal or 10 October 2021, whichever came first. The effect of carriage of HLA-DQB1*06 was estimated after adjustment for age (in years measured at baseline), healthcare worker status (defining whether individuals were healthcare workers and whether they cared for one or more than one patient with COVID-19 per week), BMI (less than 30 kg m^−^^2^ or equal to or greater than 30 kg m^−^^2^), ancestry (using the first two PCs of genetic variation to capture genetic structure) and chronic health condition (presence of none or one or more chronic respiratory, cardiovascular or diabetic health conditions). Using analyses undertaken on understanding the correlates of protection of ChAdOx1 nCoV-19 (ref. ^13^), we aimed to perform an identical modeling exercise, and, thus, samples were reweighted based on the interval between first and second vaccines (no second dose, <6, 6–8, 9–11 and ≥12 weeks) and dose arm of trial (standard dose (SD) alone, SD/SD, low dose (LD)/SD and SD/LD) distributions of individuals within the entire genotyped set with antibody data available using inverse probability weighting calculated using the ‘ipw’ package in R^54^. All analyses were undertaken in R version 4.1.1, except estimation of the genomic inflation factor (λ), which was undertaken in R version 3.6.2 using the GenABEL package^55^.

#### Replication cohort

Individuals from all three replication cohorts were stratified by HLA-DQB1*06 and DRB1-71E/R status and tested for association with log_10_-transformed anti-spike antibody levels measured after the first vaccine dose (and before the second vaccine) using linear regression adjusting for age, sex, self-reported ethnicity, priming vaccine, study and interval between prime and blood sample in days. Individuals were either analyzed together or stratified according to first vaccine dose received (ChAdOx1 nCoV-19 or BNT162b2). Survival analyses were performed using the same methods as for the discovery cohorts but including only age, sex and first vaccine type received as covariates, with reweighting performed using interval between first and second vaccines in days. Censoring was undertaken at point of breakthrough infection, withdrawal from study or date of database locking (21 January 2022 for COMCOV/COMCOV2 or 29 November 2021 for COV006), whichever came soonest. Healthcare worker status, BMI and chronic health condition information was not available for COV006, and so the variables were not included in the final replication analysis. All replication analyses were performed in R version 4.1.1.

### Reporting summary

Further information on research design is available in the Nature Portfolio Reporting Summary linked to this article.

## Online content

Any methods, additional references, Nature Portfolio reporting summaries, source data, extended data, supplementary information, acknowledgements, peer review information; details of author contributions and competing interests; and statements of data and code availability are available at 10.1038/s41591-022-02078-6.

## Supplementary information

Supplementary Information Supplementary Note, Supplementary Figs. 1–5 and Supplementary Tables 1–7

Reporting Summary

## Data Availability

The University of Oxford is committed to providing access to anonymized data for non-commercial research. Participant genotype and phenotype data will be deposited in the European Genome-phenome Archive and will be made available for non-commercial use only (accession number EGAS00001006909).
