# Supplementary material for: Human leukocyte antigen alleles associate with COVID-19 vaccine immunogenicity and risk of breakthrough infection
Source: Nat Med. 2022 Oct 13;29(1):147–57. doi: 10.1038/s41591-022-02078-6 (PMC9873562; doi:10.1038/s41591-022-02078-6)
Supplement: Supplementary file 1 — Supplementary Note, Supplementary Figs. 1–5 and Supplementary Tables 1–7 [file 41591_2022_2078_MOESM1_ESM.pdf]

nature medicine

Article https://doi.org/10.1038/s41591-022-02078-6

Human leukocyte antigen alleles associate with COVID-19 vaccine immunogenicity and risk of breakthrough infection

In the format provided by the authors and unedited

Human leukocyte antigen alleles associate with COVID-19 vaccine immunogenicity and risk of breakthrough infection

Alexander J Mentzer*, Daniel O'Connor*, Sagida Bibi, Irina Chelysheva, Elizabeth A Clutterbuck, Tesfaye Demissie, Tanya Dinesh, Nick J Edwards, Sally Felle, Shuo Feng, Amy L Flaxman, Eleanor Karp-Tatham, Grace Li, Xinxue Liu, Natalie Marchevsky, Leila Godfrey, Rebecca Makinson, Maireid B Bull, Jamie Fowler, Bana Alamad, Tomas Malinauskas, Amanda Y Chong, Katherine Sanders, Robert H Shaw, Merryn Voysey, Oxford COVID Vaccine Trial Genetics Study Team Group, Matthew D Snape, Andrew J Pollard*, Teresa Lambe*, Julian C Knight*

*Contributed equally

Supplementary Appendix

Table of Contents

Supplementary Information 2
Supplementary Note 2
  Fine mapping the likely causal locus and HLA alleles 2
Supplementary Figures 3
  Supplementary Figure 1 3
  Supplementary Figure 2 4
  Supplementary Figure 3 5
  Supplementary Figure 4 6
  Supplementary Figure 5 7
Supplementary Tables 8
  Supplementary Table 1 8
  Supplementary Table 2 9
  Supplementary Table 3 10
  Supplementary Table 4 11
  Supplementary Table 5 14
  Supplementary Table 6 15
  Supplementary Table 7 16

Supplementary Information

Supplementary Note

Fine mapping the likely causal locus and HLA alleles

The HLA allele with the most significant association with RBD antibody levels in ChAdOx1 nCov-19 vaccinated individuals was HLA-DQB1*06. This HLA-DQB1 allele is frequently inherited as part of a common haplotype with HLA-DQA1*01 and HLA-DRB1*15. If the functional HLA protein unit was causally responsible for the observed association, rather than non-allelic variants (that may impact gene expression for example), then the associations would be explained most parsimoniously if described in the structural HLA unit nomenclature. For HLA-DQ, this would include combined HLA-DQA1 and HLA-DQB1 alleles, whereas for HLA-DR, due to the monomorphic nature of HLA-DRA, it would consist of HLA-DRB1 alleles alone. Thus, all imputed chromosomal HLA-DR/DQ haplotypes were phased using PHASE that explicitly accounts for multi-allelic loci, and then models including individuals described as different haplotypic combinations were compared using the Bayesian Information Criterion (BIC). If the HLA-DQ protein unit was causally associated with the protein response the model describing the merged HLA-DQA1*01/HLA-DQB1*06 haplotype would result in a lower value BIC than either HLA-DQB1*06 or HLA-DRB1*15 alone. This effect was observed as the combined HLA-DQA1*01/HLA-DQB1*06 resulted in a BIC of 2715.2 compared to HLA-DQB1*06 alone (2715.6), HLA-DRB1*15 alone (2719.1, **Supplementary Figure 3**), supporting the likely causal variant being the functional HLA-DQ locus.

Supplementary Figures

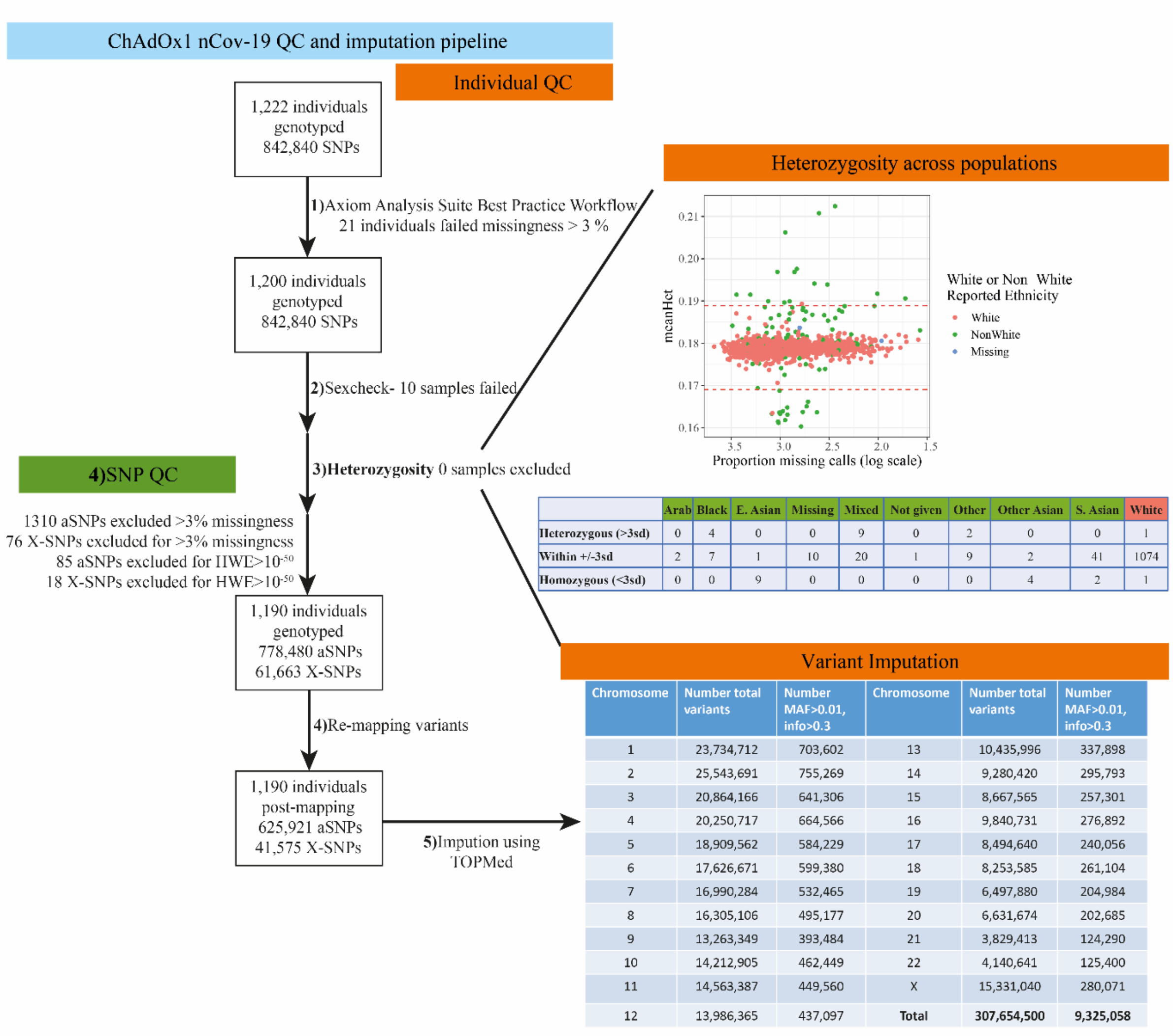

	Arab	Black	E. Asian	Missing	Mixed	Not given	Other	Other Asian	S. Asian	White
Heterozygous (>3sd)	0	4	0	0	9	0	2	0	0	1
Within +/-3sd	2	7	1	10	20	1	9	2	41	1074
Homozygous (<3sd)	0	0	9	0	0	0	0	4	2	1

Chromosome	Number total variants	Number MAF>0.01, info>0.3	Chromosome	Number total variants	Number MAF>0.01, info>0.3
1	23,734,712	703,602	13	10,435,996	337,898
2	25,543,691	755,269	14	9,280,420	295,793
3	20,864,166	641,306	15	8,667,565	257,301
4	20,250,717	664,566	16	9,840,731	276,892
5	18,909,562	584,229	17	8,494,640	240,056
6	17,626,671	599,380	18	8,253,585	261,104
7	16,990,284	532,465	19	6,497,880	204,984
8	16,305,106	495,177	20	6,631,674	202,685
9	13,263,349	393,484	21	3,829,413	124,290
10	14,212,905	462,449	22	4,140,641	125,400
11	14,563,387	449,560	X	15,331,040	280,071
12	13,986,365	437,097	**Total**	**307,654,500**	**9,325,058**

Supplementary Figure 1

Quality control of genotyped individuals from COV001 and COV002. The breakdown of individuals and SNP variants passing each stage of assessment is shown.

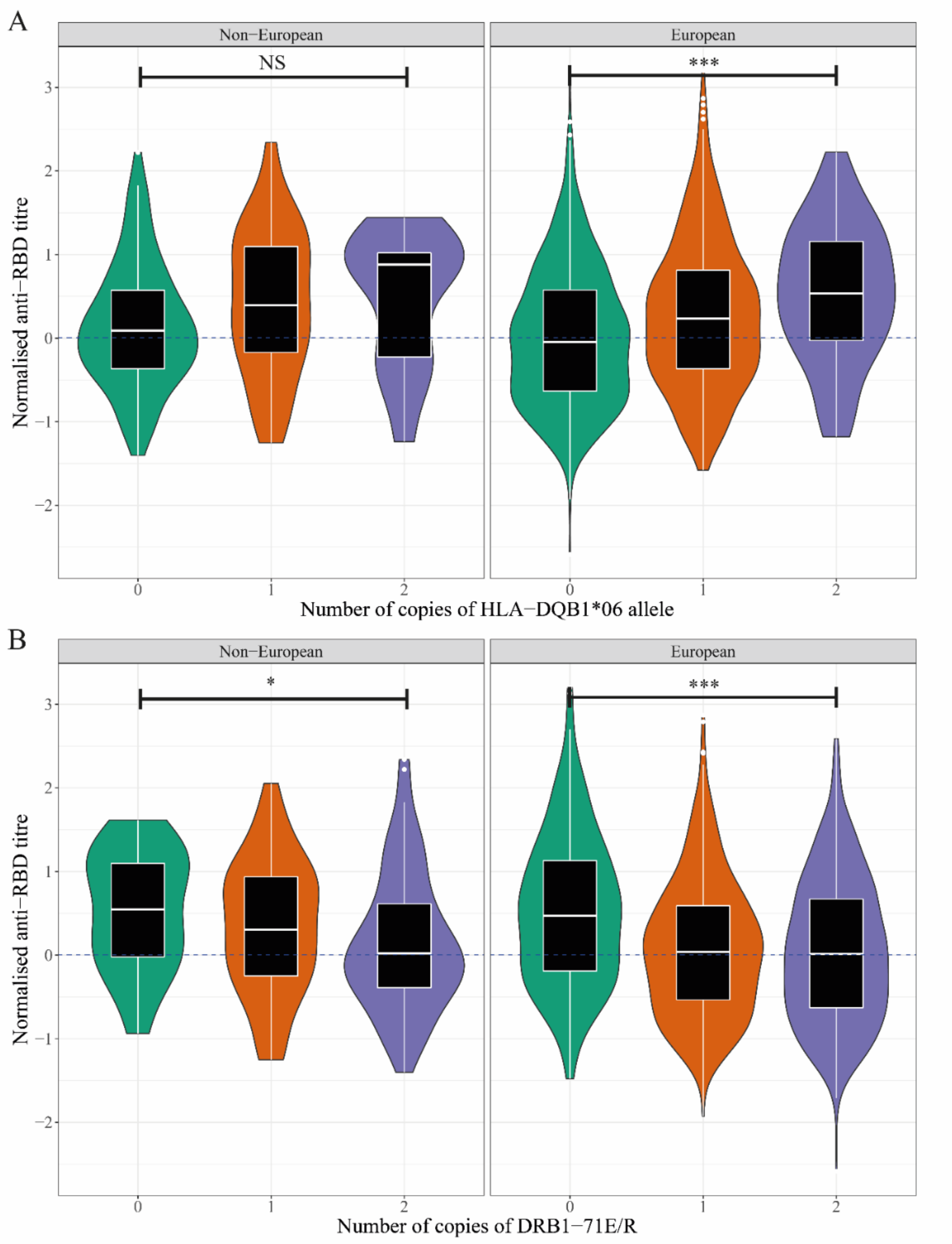

Supplementary Figure 2

Testing for evidence of the HLA associations preserved in individuals of both European and non-European ancestry in COV001 and COV002. The observed associations between carriage of HLA-DQB1*06 alleles (A) and DRB1-71E/R (B) with normalised anti-RBD titre was observed in 928 European individuals and 148 non-European individuals using unadjusted linear regression and tested for differences between groups using two-sided Student's t-tests. Box plot center line, median; box limits, upper and lower quartiles; whiskers, 1.5x interquartile range. ***$P<1x10^{-3}$, *P<0.05, NS not significant.

Allele or haplotype	BIC
DQB1*06 alone	2715.6
DRB1*15 alone	2719.1
DQA1*01 and DQB1*06	2715.2
DRB1*15 and no DQB1*06	2749.7
DQB1*06 and no DRB1*15	2746.2
DRB1*15 and DQB1*06	2715.4

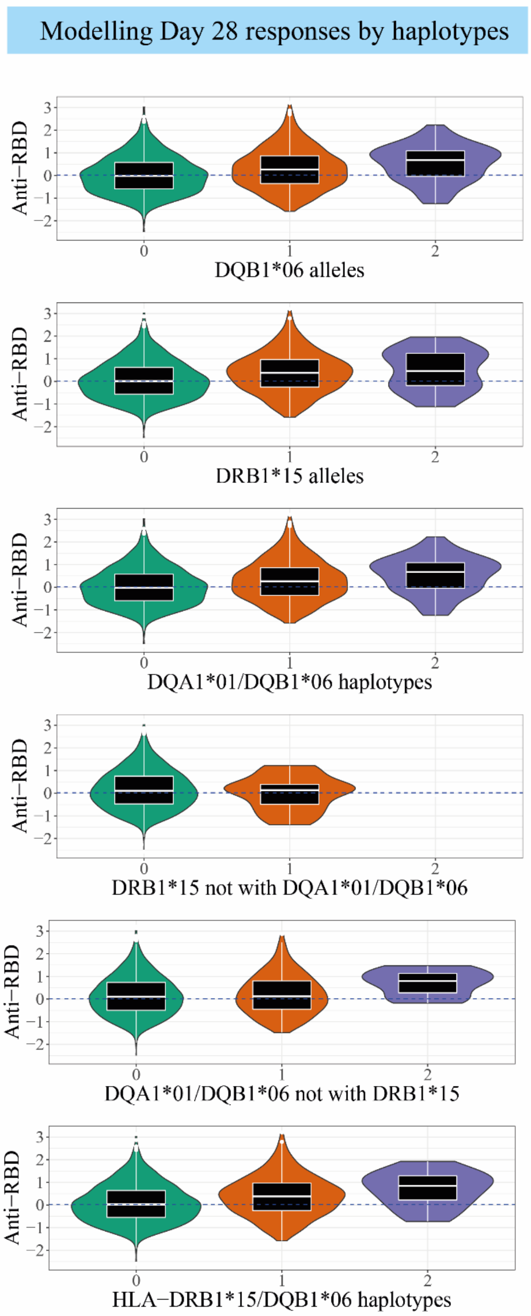

Supplementary Figure 3

Modelling allelic associations to fine-map likely HLA causal locus in 1,023 COV001 and COV002 individuals restricted by self-reported White ethnicity and PCA axes, and with IBD values less than or equal to 0.185. Bayesian information criterion modelling was used to test the most parsimonious fit with RBD levels using phased HLA haplotypes adjusting for age, sex, assay laboratory (MSD or PPD) and five genetic principal components. Box plot center line, median; box limits, upper and lower quartiles; whiskers, 1.5x interquartile range.

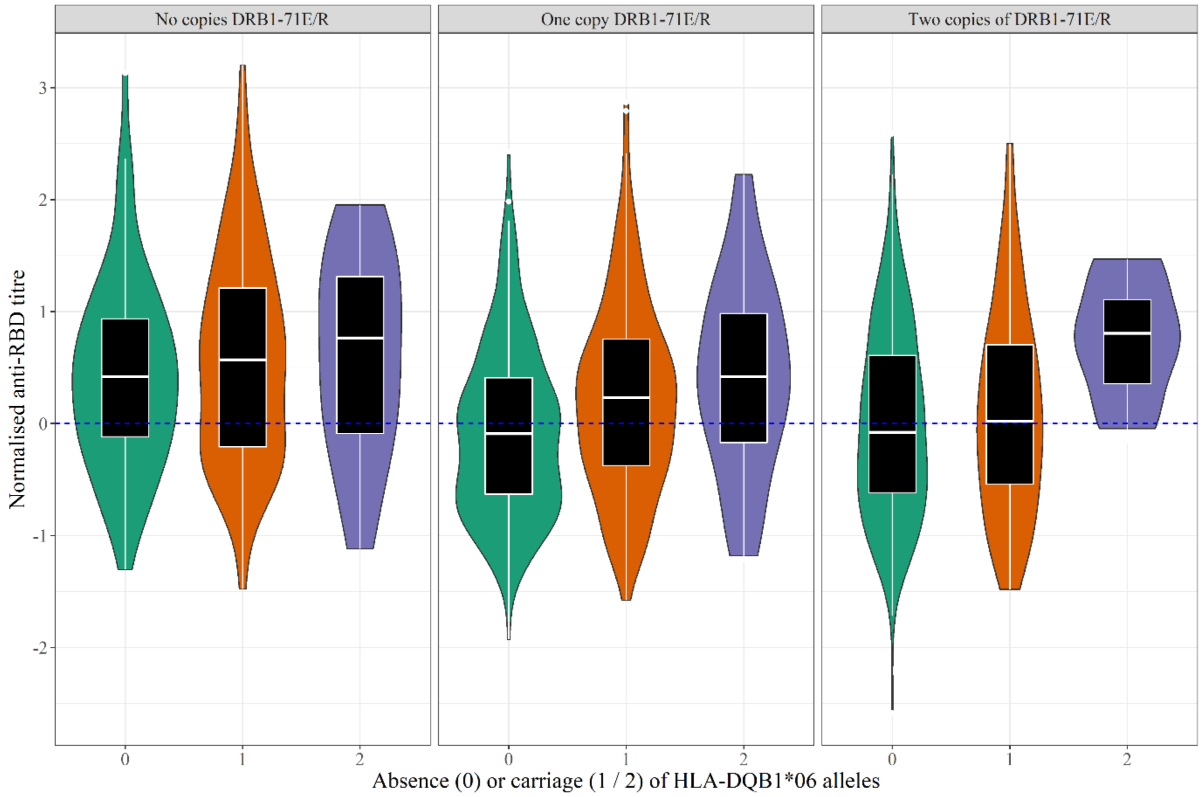

Supplementary Figure 4

Assessing inter-dependence of two associations across the MHC with RBD antibody levels in 1,076 participants from COV001 and COV002: HLA-DQB1*06 and DRB1-71E/R. The distribution of antibody levels in individuals carrying 0, 1 or 2 copies of HLA-DQB1*06 stratified by the number of residues of DRB1-71E/R carried. Box plot center line, median; box limits, upper and lower quartiles; whiskers, 1.5x interquartile range.

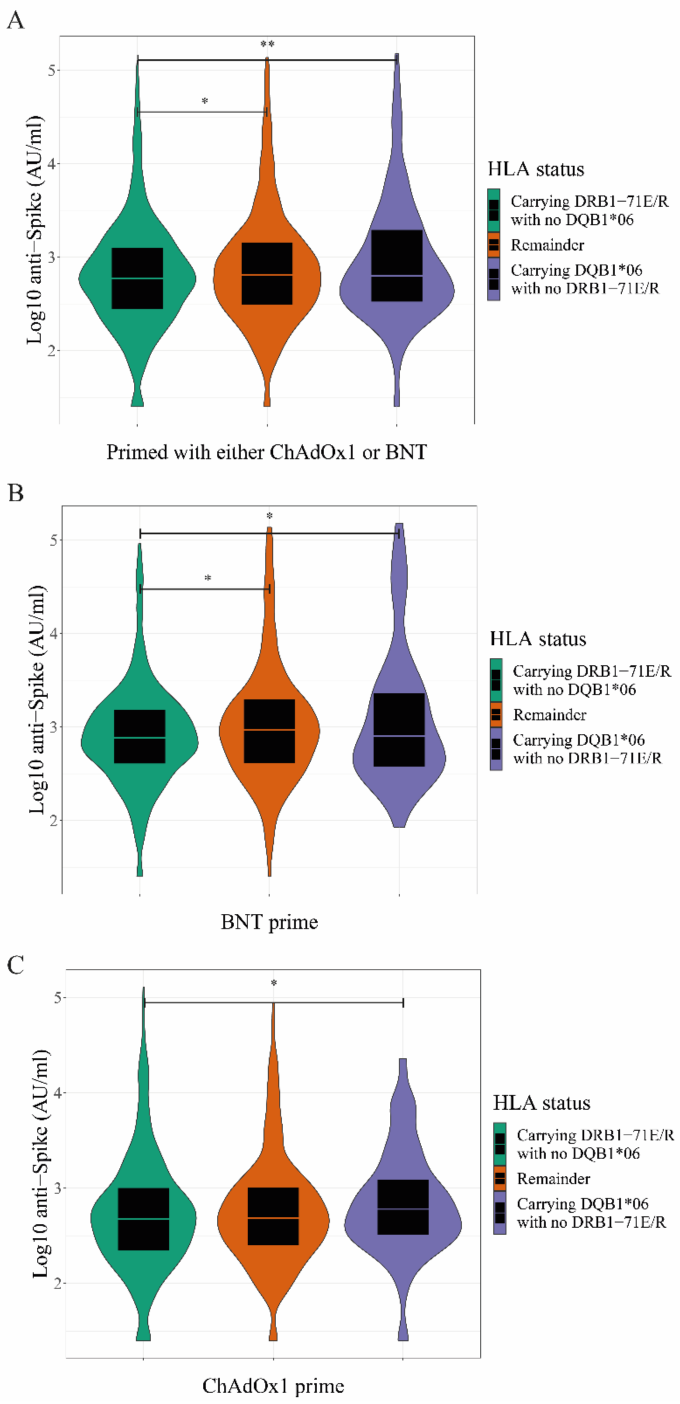

Supplementary Figure 5

Independent replication of the HLA association with response to COVID vaccination. A) The association replicated in an independently recruited set of 1,677 individuals from three clinical trials: COMCOV, COMCOV2 and COV006, irrespective of the vaccine used for priming. The same associations were observed both for the 746 individuals primed with BNT162b2, (B), and 931 primed with ChAdOx1 (C). Statistical tests are linear regression adjusting for age, sex, self-reported ethnicity, priming vaccine, study and interval between prime and blood sample in days. Box plot center line, median; box limits, upper and lower quartiles; whiskers, 1.5x interquartile range ** P<0.01; * P<0.05.

Supplementary Tables

Supplementary Table 1

Imputed HLA alleles associated with RBD antibody levels in COV001 and COV002 individuals restricted by IBD<0.185, self-reported White ethnicity and PC cutoffs. Alleles with minor allele frequencies greater than 0.01 and *P* values (calculated using linear regression) less than 0.01 are shown.

HLA allele	Minor allele frequency	Beta	SE	*P*
HLA-DQB1*06	0.25	0.27	0.04	$3.2x10^{-9}$
HLA-DQA1*01:02:01:01	0.19	0.28	0.05	$1.3x10^{-8}$
HLA-DRB1*15:01:01:01	0.14	0.31	0.05	$1.8x10^{-8}$
HLA-DQB1*06:02:01:01	0.14	0.31	0.05	$3.2x10^{-8}$
HLA-DRB1*15	0.17	0.28	0.05	$2.1x10^{-7}$
HLA-DPB1*04:01:01:01	0.44	0.15	0.04	$1.7x10^{-4}$
HLA-DQB1*05	0.15	-0.18	0.05	$1.0x10^{-3}$
HLA-C*04	0.09	-0.23	0.07	$1.0x10^{-3}$
HLA-C*04:01:01:01	0.09	-0.23	0.07	$1.0x10^{-3}$
HLA-DPB1*04:02:01:01	0.11	-0.18	0.06	$1.8x10^{-3}$
HLA-DQA1*01:01:01:01	0.14	-0.17	0.06	$2.4x10^{-3}$
HLA-C*07	0.34	0.12	0.04	$3.7x10^{-3}$
HLA-DQA1*02	0.14	-0.16	0.06	$4.1x10^{-3}$
HLA-DQA1*02:01:01:01	0.14	-0.16	0.06	$4.1x10^{-3}$
HLA-C*07:02:01:01	0.15	0.16	0.05	$4.3x10^{-3}$
HLA-DRB1*07:01:01:01	0.13	-0.16	0.06	$5.5x10^{-3}$
HLA-DQA1*01	0.14	0.11	0.04	$6.4x10^{-3}$
HLA-DRB1*07	0.14	-0.15	0.06	$6.9x10^{-3}$
HLA-DRB1*13	0.10	0.17	0.06	$7.4x10^{-3}$
HLA-B*08	0.13	0.15	0.06	$8.0x10^{-3}$
HLA-B*08:01:01:01	0.13	0.15	0.06	$8.0x10^{-3}$
HLA-DRB1*13:02:01:01	0.04	0.26	0.09	$5.4x10^{-3}$

Supplementary Table 2

Imputed HLA alleles associated with S antibody levels in COV001 and COV002. Alleles with minor allele frequencies greater than 0.01 and *P* values (calculated using linear regression) less than 0.01 are shown.

HLA allele	Minor allele frequency	Beta	SE	*P*
HLA-DRB1*15:01:01:01	0.14	0.17	0.03	$4.6x10^{-8}$
HLA-DQA1*01:02:01:01	0.19	0.15	0.03	$7.8x10^{-8}$
HLA-DQB1*06:02:01:01	0.14	0.17	0.03	$9.4x10^{-8}$
HLA-DQB1*06	0.25	0.13	0.03	$2.0x10^{-7}$
HLA-DRB1*15	0.17	0.15	0.03	$9.2x10^{-7}$
HLA-DPB1*04:01:01:01	0.44	0.08	0.02	$6.5x10^{-4}$
HLA-C*07:02:01:01	0.15	0.10	0.03	$1.6x10^{-3}$
HLA-C*07	0.34	0.07	0.02	$2.2x10^{-3}$
HLA-B*44:03:01:01	0.05	-0.15	0.05	$2.6x10^{-3}$
HLA-DPB1*04:02:01:01	0.11	-0.10	0.03	$2.7x10^{-3}$
HLA-DQA1*01	0.40	0.06	0.02	$4.5x10^{-3}$
HLA-B*44	0.16	-0.08	0.03	$5.4x10^{-3}$
HLA-DRB1*07	0.14	-0.09	0.03	$6.2x10^{-3}$
HLA-B*08	0.13	0.08	0.03	$8.7x10^{-3}$
HLA-B*08:01:01:01	0.13	0.08	0.03	$8.7x10^{-3}$

Supplementary Table 3

Concordance between HLA alleles in 60 individuals with both genotype data and classical HLA type data available from COV001 and COV002 trials. HLA alleles were imputed from genotype data.

Locus	Number of alleles	2-digit concordance	4-digit concordance
A	120	1.00	0.97
B	120	1.00	0.94
C	120	1.00	0.97
DQB1	120	0.99	0.82
DRB1	120	0.97	0.92

Supplementary Table 4

Performance statistics of HLA imputation in 60 individuals with both imputed data and classically typed allele data available in COV001 and COV002, distributed by locus and allele.

Locus	Allele*	Number observed in classically typed set	Number observed in imputed set	Sensitivity	Specificity	PPV	NPV	Accuracy
A	0101	16	16	1.00	1.00	1.00	1.00	1.00
A	0201	35	34	0.94	0.99	0.97	0.98	0.98
A	0202	0	1	NA	NA	NA	NA	NA
A	0205	1	1	1.00	1.00	1.00	1.00	1.00
A	0301	16	16	1.00	1.00	1.00	1.00	1.00
A	1101	12	12	1.00	1.00	1.00	1.00	1.00
A	2301	1	1	1.00	1.00	1.00	1.00	1.00
A	2402	11	11	1.00	1.00	1.00	1.00	1.00
A	2501	3	3	1.00	1.00	1.00	1.00	1.00
A	2601	6	6	1.00	1.00	1.00	1.00	1.00
A	2901	1	0	NA	NA	NA	NA	NA
A	2902	1	2	1.00	0.99	0.50	1.00	0.99
A	3001	2	2	1.00	1.00	1.00	1.00	1.00
A	3002	2	1	0.50	1.00	1.00	0.99	0.99
A	3101	3	3	1.00	1.00	1.00	1.00	1.00
A	3201	3	3	1.00	1.00	1.00	1.00	1.00
A	3303	1	1	1.00	1.00	1.00	1.00	1.00
A	6601	2	2	1.00	1.00	1.00	1.00	1.00
A	6801	1	0	NA	NA	NA	NA	NA
A	6802	2	2	1.00	1.00	1.00	1.00	1.00
A	6803	1	1	1.00	1.00	1.00	1.00	1.00
B	0702	13	13	1.00	1.00	1.00	1.00	1.00
B	0801	4	4	1.00	1.00	1.00	1.00	1.00
B	1302	3	3	1.00	1.00	1.00	1.00	1.00
B	1401	1	1	1.00	1.00	1.00	1.00	1.00
B	1402	8	8	1.00	1.00	1.00	1.00	1.00
B	1501	7	7	1.00	1.00	1.00	1.00	1.00
B	1517	1	1	1.00	1.00	1.00	1.00	1.00
B	1801	3	3	1.00	1.00	1.00	1.00	1.00
B	2704	4	0	NA	NA	NA	NA	NA
B	2705	5	9	1.00	0.97	0.56	1.00	0.97
B	3501	10	8	0.70	0.99	0.88	0.97	0.97
B	3502	1	1	1.00	1.00	1.00	1.00	1.00
B	3503	2	3	1.00	0.99	0.67	1.00	0.99
B	3701	2	2	1.00	1.00	1.00	1.00	1.00
B	3801	4	4	1.00	1.00	1.00	1.00	1.00
B	3901	1	1	1.00	1.00	1.00	1.00	1.00
B	3905	1	1	1.00	1.00	1.00	1.00	1.00
B	4001	4	4	1.00	1.00	1.00	1.00	1.00
B	4002	2	2	1.00	1.00	1.00	1.00	1.00

B	4101	3	2	0.67	1.00	1.00	0.99	0.99
B	4102	0	1	NA	NA	NA	NA	NA
B	4402	14	14	1.00	1.00	1.00	1.00	1.00
B	4403	6	6	1.00	1.00	1.00	1.00	1.00
B	4501	1	1	1.00	1.00	1.00	1.00	1.00
B	4901	1	1	1.00	1.00	1.00	1.00	1.00
B	5001	2	2	1.00	1.00	1.00	1.00	1.00
B	5101	3	3	1.00	1.00	1.00	1.00	1.00
B	5401	1	1	1.00	1.00	1.00	1.00	1.00
B	5501	3	3	1.00	1.00	1.00	1.00	1.00
B	5601	1	1	1.00	1.00	1.00	1.00	1.00
B	5701	7	7	1.00	1.00	1.00	1.00	1.00
B	5801	2	2	1.00	1.00	1.00	1.00	1.00
C	0102	9	9	1.00	1.00	1.00	1.00	1.00
C	0202	6	6	1.00	1.00	1.00	1.00	1.00
C	0302	1	1	1.00	1.00	1.00	1.00	1.00
C	0303	8	8	1.00	1.00	1.00	1.00	1.00
C	0304	6	6	1.00	1.00	1.00	1.00	1.00
C	0401	17	16	0.94	1.00	1.00	0.99	0.99
C	0407	0	1	NA	NA	NA	NA	NA
C	0501	14	14	1.00	1.00	1.00	1.00	1.00
C	0602	13	13	1.00	1.00	1.00	1.00	1.00
C	0701	11	9	0.82	1.00	1.00	0.98	0.98
C	0702	13	15	1.00	0.98	0.87	1.00	0.98
C	0802	9	9	1.00	1.00	1.00	1.00	1.00
C	1202	1	0	NA	NA	NA	NA	NA
C	1203	5	6	1.00	0.99	0.83	1.00	0.99
C	1402	1	1	1.00	1.00	1.00	1.00	1.00
C	1502	1	1	1.00	1.00	1.00	1.00	1.00
C	1601	3	3	1.00	1.00	1.00	1.00	1.00
C	1701	2	2	1.00	1.00	1.00	1.00	1.00
DQB1	0201	10	25	0.90	0.85	0.36	0.99	0.86
DQB1	0202	14	0	NA	NA	NA	NA	NA
DQB1	0301	25	24	0.92	0.99	0.96	0.98	0.98
DQB1	0302	10	10	1.00	1.00	1.00	1.00	1.00
DQB1	0303	7	7	1.00	1.00	1.00	1.00	1.00
DQB1	0401	2	1	0.50	1.00	1.00	0.99	0.99
DQB1	0402	1	2	1.00	0.99	0.50	1.00	0.99
DQB1	0501	18	17	0.89	0.99	0.94	0.98	0.98
DQB1	0502	0	1	NA	NA	NA	NA	NA
DQB1	0503	3	3	1.00	1.00	1.00	1.00	1.00
DQB1	0602	18	14	0.56	0.96	0.71	0.92	0.90
DQB1	0603	4	5	1.00	0.99	0.80	1.00	0.99
DQB1	0604	5	6	1.00	0.99	0.83	1.00	0.99
DQB1	0605	1	0	NA	NA	NA	NA	NA
DQB1	0609	2	5	1.00	0.97	0.40	1.00	0.98
DRB1	0101	13	13	1.00	1.00	1.00	1.00	1.00
DRB1	0102	1	1	1.00	1.00	1.00	1.00	1.00

DRB1	0103	2	3	1.00	0.99	0.67	1.00	0.99
DRB1	0301	11	11	1.00	1.00	1.00	1.00	1.00
DRB1	0401	10	9	0.90	1.00	1.00	0.99	0.99
DRB1	0402	1	1	1.00	1.00	1.00	1.00	1.00
DRB1	0403	2	2	0.50	0.99	0.50	0.99	0.98
DRB1	0404	2	1	0.00	0.99	0.00	0.98	0.98
DRB1	0405	3	3	1.00	1.00	1.00	1.00	1.00
DRB1	0407	3	3	0.67	0.99	0.67	0.99	0.98
DRB1	0408	1	1	1.00	1.00	1.00	1.00	1.00
DRB1	0701	19	19	1.00	1.00	1.00	1.00	1.00
DRB1	0801	2	3	1.00	0.99	0.67	1.00	0.99
DRB1	0901	0	1	NA	NA	NA	NA	NA
DRB1	1101	6	4	0.50	0.99	0.75	0.97	0.97
DRB1	1102	2	1	0.50	1.00	1.00	0.99	0.99
DRB1	1103	0	1	NA	NA	NA	NA	NA
DRB1	1104	1	1	0.00	0.99	0.00	0.99	0.98
DRB1	1201	3	3	1.00	1.00	1.00	1.00	1.00
DRB1	1301	5	5	1.00	1.00	1.00	1.00	1.00
DRB1	1302	11	11	1.00	1.00	1.00	1.00	1.00
DRB1	1303	3	3	1.00	1.00	1.00	1.00	1.00
DRB1	1305	0	1	NA	NA	NA	NA	NA
DRB1	1401	4	3	0.75	1.00	1.00	0.99	0.99
DRB1	1501	13	13	1.00	1.00	1.00	1.00	1.00
DRB1	1503	1	1	1.00	1.00	1.00	1.00	1.00
DRB1	1601	1	1	1.00	1.00	1.00	1.00	1.00

*Alleles provided in 4-digit format. e.g. 0101 in locus A represents HLA-A*01:01

Supplementary Table 5

Baseline characteristics for 1,076 participants who received ChAdOx1 nCoV-19 vaccine with genotype data available in COV001 and COV002 (by HLA-DQB1*06 strata).

		Not accounting for DRB1-71E/R		Accounting for DRB1-71E/R		
**Characteristic (range where applicable)**	**Genotyped cohort (ChAdOx1 vaccinated) N=1,076**	**HLA-DQB1*06 carrier (N=474)**	**Non HLA-DQB1*06 carrier (N=602)**	**Carrying DRB1-71E/R with no DQB1*06 (N=532)**	**Remainder (N=419)**	**Carrying DQB1*06 with no DRB1-71E/R (N=125)**
**Age at recruitment**	37.0 (30.0-47.0)	37.0 (29.0-46.0)	38.0 (30.0-47.0)	38.0 (30.0-47.0)	37.0 (29.0-47.0)	38.0 (29.0-45.6)
18-55yrs, number (%)	1076 (100)	474 (100)	602 (100)	532 (100)	419 (100)	125 (100)
Missing, number (%)	0 (0)	0 (0)	0 (0)	0 (0)	0 (0)	0 (0)
**Sex**, number (%)						
Female	572 (53.2)	255 (53.8)	317 (52.7)	281 (52.8)	218 (52.0)	73 (58.4)
Male	504 (46.8)	219 (46.2)	285 (47.3)	251 (47.2)	201 (48.0)	52 (41.6)
**BMI**						
Median (IQR)	24.7 (22.6-27.5)	24.7 (22.6-27.4)	24.7 (22.5–27.7)	24.7 (22.5-27.52)	25.0 (22.6-27.5)	24.4 (22.5-27.4)
**Ethnic group**, number (%)						
White	974 (90.5)	439 (92.6)	535 (88.8)	469 (88.2)	385 (91.9)	120 (96.0)
Asian	50 (4.7)	14 (3.0)	36 (6.0)	32 (6.0)	16 (3.8)	2 (1.6)
Black	10 (0.9)	9 (1.9)	1 (0.2)	1 (0.2)	7 (1.7)	2 (1.6)
Mixed	29 (2.7)	11 (2.3)	18 (3.0)	18 (3.4)	10 (2.4)	1 (0.8)
Other	13 (1.2)	1 (0.2)	12 (2.0)	12 (2.2)	1 (0.2)	0 (0)
Not reported / missing	0 (0)	0 (0)	0 (0)	0 (0)	0 (0)	0 (0)
**Health and social care setting workers (HCW)**, number (%)						
Not HCW	590 (54.8)	259 (54.7)	331 (55.0)	301 (56.6)	225 (53.7)	64 (51.2)
HCW unknown COVID contacts	101 (9.4)	50 (10.5)	51 (8.5)	44 (8.3)	44 (10.5)	13 (10.4)
HCW with ≤1 COVID contacts	267 (24.8)	115 (24.3)	152 (25.2)	130 (24.4)	101 (24.1)	36 (28.8)
HCW with >1 COVID contacts	118 (11.0)	50 (10.5)	68 (11.3)	57 (10.7)	49 (11.7)	12 (9.6)
**Comorbidities**, number (%)						
Cardiovascular	35 (3.3)	15 (3.2)	20 (3.3)	16 (3.0)	15 (3.6)	4 (3.2)
Respiratory	73 (6.8)	32 (6.8)	41 (6.8)	38 (7.1)	23 (5.5)	12 (9.6)
Diabetes	9 (0.8)	1 (0.2)	8 (1.3)	4 (0.8)	5 (1.2)	0 (0)
**Prime boost interval**, number (%)						
<6 weeks	12 (1.1)	5 (1.1)	7 (1.2)	7 (1.3)	3 (0.7)	2 (1.6)
6-8 weeks	151 (14.0)	67 (14.1)	84 (14.0)	69 (13.0)	68 (16.2)	14 (11.2)
9-11 weeks	257 (23.9)	104 (21.9)	153 (25.4)	135 (25.4)	96 (22.9)	26 (20.8)
≥12 weeks	556 (51.7)	257 (54.2)	299 (49.6)	269 (50.5)	209 (49.9)	78 (62.4)
none	100 (9.3)	41 (8.7)	59 (9.8)	52 (9.8)	43 (10.3)	5 (4.0)
**Arm of trial**, number (%)						
SD	499 (46.4)	223 (47.1)	276 (45.8)	248 (46.6)	191 (45.6)	60 (48.0)
SD / SD	400 (37.2)	183 (38.6)	217 (36.1)	187 (35.2)	162 (38.7)	51 (40.8)
LD / SD	167 (15.5)	64 (13.5)	103 (17.1)	91 (17.1)	63 (15.0)	13 (10.4)
SD / LD	10 (0.9)	4 (0.8)	6 (1.0)	6 (1.1)	3 (0.7)	1 (0.8)

Supplementary Table 6

Baseline characteristics for 1,677 participants from COMCOV, COMCOV2 and COV006 trials with genotype data available for replication.

Characteristic (range where applicable)	Genotyped replication cohort N=1,677	COMCOV (N=627)	COMCOV2 (N=847)	COV006 (N=203)
**Age at recruitment**, number (%)	59.7 (54.0-64.6)	57.5 (54.1-61.3)	63.7 (59.2-67.1)	12.7 (9.9-15.3)
<18yrs	203 (12.1)	0 (0)	0 (0)	203 (100)
18-55yrs	293 (17.5)	203 (32.4)	90 (10.6)	0 (0)
>55yrs	1179 (70.3)	424 (67.6)	755 (89.2)	0 (0)
Missing	2 (0.1)	0 (0)	2 (0.2)	0 (0)
**Sex**, number (%)				
Female	709 (42.3)	272 (43.4)	345 (40.7)	92 (45.3)
Male	968 (57.7)	355 (56.6)	502 (59.3)	111 (54.7)
**BMI***				
Median (IQR)	26.3 (23.7-29.6)	26.6 (23.8-29.9)	26.2 (23.7-29.4)	NA
**Ethnic group**, number (%)				
White	1462 (87.2)	481 (76.7)	798 (94.2)	183 (90.1)
Asian	105 (6.2)	77 (12.3)	24 (2.9)	4 (2.0)
Black	15 (0.9)	8 (1.3)	7 (0.8)	0 (0)
Mixed	75 (4.5)	49 (7.8)	10 (1.2)	16 (7.9)
Other	20 (1.2)	12 (1.9)	8 (0.9)	0 (0)
**Health and social care setting workers (HCW)**, number (%)*				
Not HCW	1427 (96.8)	607 (96.8)	820 (96.8)	NA
HCW unknown COVID contacts	47 (3.2)	20 (3.2)	27 (3.2)	NA
**Comorbidities**, number (%)*				
Cardiovascular	385 (26.1)	135 (21.5)	250 (29.5)	NA
Respiratory	204 (13.8)	90 (14.4)	114 (13.5)	NA
Diabetes	103 (7.0)	28 (4.5)	75 (8.9)	NA
**Time between prime and sample**, number (%)				
<6 weeks	400 (23.9)	341 (54.4)	1 (0.1)	58 (28.6)
6-8 weeks	0 (0)	0 (0)	0 (0)	0 (0)
9-11 weeks	777 (46.3)	0 (0)	777 (91.7)	0 (0)
≥12 weeks	500 (29.8)	286 (45.6)	69 (8.2)	145 (71.4)
**Priming vaccine**, number (%)				
ChAdOx1	931 (55.5)	304 (48.5)	424 (50.1)	203 (100)
BNT162b2	746 (44.5)	323 (51.5)	423 (49.9)	0 (0)
**Boosting vaccine**, number (%)				
ChAdOx1	643 (38.6)	308 (49.1)	142 (16.8)	203 (100)
BNT162b2	458 (27.5)	319 (50.9)	139 (16.4)	0 (0)
mRNA-1273	289 (17.3)	0 (0)	289 (34.1)	0 (0)
NVXCoV2373	277 (16.6)	0 (0)	277 (32.7)	0 (0)
**HLA carrier**, number (%)				
HLA-DQB1*06	715 (42.6)	272 (43.4)	359 (42.4)	84 (41.4)
DRB1-71E/R	1393 (83.1)	524 (83.6)	700 (82.6)	169 (83.3)

*Frequencies for 'replication cohort' calculated only using COMCOV and COMCOV2.

Supplementary Table 7

Baseline characteristics for 1,076 participants who received ChAdOx1 nCoV-19 vaccine with genotype data available in COV001 and COV002, and stratified by breakthrough infection category in the 1,069 individuals who experienced breakthrough infection 21 days or more after receiving the first vaccine.

Characteristics	All	Negative	Primary	Non-primary	Asymptomatic
**N enrolled**	1076	957	41	8	63
**Sex (male)**, number (%)	504 (46.8)	443 (46.3)	20 (48.8)	7 (87.5)	29 (46.0)
**Age at recruitment** (Years), Median (IQR)	37.0 (30.0-47.0)	37.0 (30.0-47.0)	35.0 (28.0-48.0)	32.1 (26.5-40.0)	40.0 (30.0-47.5)
**BMI**, Median (IQR)	24.7 (22.5-27.5)	24.7 (22.5-27.5)	24.9 (23.1-27.5)	26.1 (24.1-27.3)	25.4 (22.6-28.8)
**Comorbidities**, number (%)					
Respiratory disease	73 (6.8)	59 (6.2)	5 (12.2)	1 (12.5)	8 (12.7)
Cardiovascular disease	35 (3.3)	29 (3.0)	2 (4.9)	3 (37.5)	1 (1.6)
Diabetes	9 (0.8)	7 (0.7)	2 (4.9)	0 (0)	0 (0)
**Ethnic group**, number (%)					
White	974 (90.5)	865 (90.4)	37 (90.2)	7 (87.5)	58 (92.1)
Asian	50 (4.6)	44 (4.6)	1 (2.4)	1 (12.5)	4 (6.3)
Black	10 (1.0)	9 (0.9)	0 (0)	0 (0)	1 (1.6)
Mixed	29 (2.7)	26 (2.7)	3 (7.3)	0 (0)	0 (0)
Other	13 (1.2)	13 (1.4)	0 (0)	0 (0)	0 (0)
**Health and social care setting workers (HCW)**, number (%)					
Not HCW	590 (54.8)	539 (56.3)	19 (46.3)	3 (37.5)	26 (41.3)
HCW unknown COVID contacts	101 (9.4)	91 (9.5)	6 (14.6)	1 (12.5)	3 (4.8)
HCW with ≤1 COVID contacts	267 (24.8)	226 (23.6)	10 (24.4)	3 (37.5)	27 (42.8)
HCW with >1 COVID contacts	118 (11)	101 (10.6)	6 (14.6)	1 (12.5)	7 (11.1)
**Prime boost interval**, number (%)					
<6 weeks	12 (1.1)	11 (1.1)	0 (0)	0 (0)	1 (1.6)
6-8 weeks	151 (14)	133 (13.9)	7 (17.1)	1 (12.5)	9 (14.3)
9-11 weeks	257 (23.9)	220 (23.0)	13 (31.7)	2 (25.0)	18 (28.6)
≥12 weeks	556 (51.7)	501 (52.4)	15 (36.6)	5 (62.5)	33 (52.4)
none	100 (9.3)	92 (9.6)	6 (14.6)	0 (0)	2 (3.1)
**Arm of trial**, number (%)					
SD	499 (46.4)	463 (48.4)	18 (43.9)	1 (12.5)	16 (25.4)
SD / SD	400 (37.2)	342 (35.8)	17 (41.5)	6 (75.0)	31 (49.2)
LD / SD	167 (15.5)	143 (14.9)	6 (14.6)	1 (12.5)	15 (23.8)
SD / LD	10 (0.9)	9 (0.9)	0 (0)	0	1 (1.6)
**HLA-DQB1 carrier**, number (%)					
HLA-DQB1*06	474 (44.1)	436 (45.5)	13 (31.7)	6 (75.0)	19 (30.2)
DRB1-71E/R	881 (81.9)	784 (81.9)	37 (90.2)	7 (87.5)	51 (76.2)
