# Supplementary material for: Human leukocyte antigen alleles associate with COVID-19 vaccine immunogenicity and risk of breakthrough infection
Source: Nat Med. 2022 Oct 13;29(1):147–57. doi: 10.1038/s41591-022-02078-6 (PMC9873562; doi:10.1038/s41591-022-02078-6)
Supplement: Supplementary file 2 — Reporting Summary [file 41591_2022_2078_MOESM2_ESM.pdf]

nature portfolio

Corresponding author(s): Mentzer

Last updated by author(s): Sep 23, 2022

Reporting Summary

Nature Portfolio wishes to improve the reproducibility of the work that we publish. This form provides structure for consistency and transparency in reporting. For further information on Nature Portfolio policies, see our Editorial Policies and the Editorial Policy Checklist.

Statistics

For all statistical analyses, confirm that the following items are present in the figure legend, table legend, main text, or Methods section.

n/a	Confirmed	
☐	☒	The exact sample size (*n*) for each experimental group/condition, given as a discrete number and unit of measurement
☐	☒	A statement on whether measurements were taken from distinct samples or whether the same sample was measured repeatedly
☐	☒	The statistical test(s) used AND whether they are one- or two-sided<br>*Only common tests should be described solely by name; describe more complex techniques in the Methods section.*
☐	☒	A description of all covariates tested
☐	☒	A description of any assumptions or corrections, such as tests of normality and adjustment for multiple comparisons
☐	☒	A full description of the statistical parameters including central tendency (e.g. means) or other basic estimates (e.g. regression coefficient) AND variation (e.g. standard deviation) or associated estimates of uncertainty (e.g. confidence intervals)
☐	☒	For null hypothesis testing, the test statistic (e.g. *F*, *t*, *r*) with confidence intervals, effect sizes, degrees of freedom and *P* value noted<br>*Give P values as exact values whenever suitable.*
☐	☒	For Bayesian analysis, information on the choice of priors and Markov chain Monte Carlo settings
☐	☒	For hierarchical and complex designs, identification of the appropriate level for tests and full reporting of outcomes
☐	☒	Estimates of effect sizes (e.g. Cohen's *d*, Pearson's *r*), indicating how they were calculated

Our web collection on statistics for biologists contains articles on many of the points above.

Software and code

Policy information about availability of computer code

Data collection	PLINK (version 1.9, https://www.cog-genomics.org/plink/)<br>Multi-Ethnic HLA reference panel (version 1.0, 2021, https://imputationserver.readthedocs.io/en/latest/reference-panels/)
Data analysis	PLINK (version 1.9, https://www.cog-genomics.org/plink/)<br>DosageConverter (version 1.0.4, https://genome.sph.umich.edu/wiki/DosageConvertor)<br>PHASE (version 2.1.1, https://stephenslab.uchicago.edu/phase/download.html)<br>AlphaFold (https://colab.research.google.com/github/sokrypton/ColabFold/blob/main/AlphaFold2.ipynb)<br>PyMOL Molecular Graphics System (version 2.3.2, https://pymol.org/2/)<br>GCTA (version 1.24.4, https://yanglab.westlake.edu.cn/software/gcta/#Overview)<br>R (version 4.1.1; packages: ipw, ggplot2, survminer, survival, perm,)<br>R (version 3.6.2; GenABEL package)

For manuscripts utilizing custom algorithms or software that are central to the research but not yet described in published literature, software must be made available to editors and reviewers. We strongly encourage code deposition in a community repository (e.g. GitHub). See the Nature Portfolio guidelines for submitting code & software for further information.

Data

Policy information about availability of data

All manuscripts must include a data availability statement. This statement should provide the following information, where applicable:

- Accession codes, unique identifiers, or web links for publicly available datasets
- A description of any restrictions on data availability
- For clinical datasets or third party data, please ensure that the statement adheres to our policy

The datasets generated during and/or analysed during the current study are available from the corresponding author on reasonable request.

Human research participants

Policy information about studies involving human research participants and Sex and Gender in Research.

Reporting on sex and gender	The biological attribute of sex was used in this study. The justification for this is that biological sex was used as sample quality control metric, and the sex attribute used in analysis was derived from the genetic data.
Population characteristics	**Discovery cohort**<br>The participants were enrolled in phase 1/2 (COV001) or phase 2/3 (COV002) randomized single-blind ChAdOx1 nCoV-19 (AZD1222) vaccine multi-centre efficacy trials conducted across multiple sites within the United Kingdom (NCT04324606, NCT04400838). In brief, following written informed consent adults aged 18 years and older were randomly assigned to receive either intramuscular ChAdOx1 nCoV-19 (AZD1222) or a control vaccine (MenACWY), to assess the safety and efficacy of the ChAdOx1 nCoV-19 vaccine against SARS-CoV-2.<br><br>** Replication cohort **<br>The replication cohort was comprised of participants from three COVID-19 vaccine trials conducted across several sites within the United Kingdom. Two of these trials (COMCOV and COMCOV2) were in adults aged 50 years and older, randomised to receive homologous or heterologous two-dose schedules of either intramuscular ChAdOx1 nCoV-19, mRNA vaccines (BNT162b2 or mRNA-1273) or a nanoparticle vaccine (NVX-CoV2373). The other trial (COV006) was in children aged 6-17 who were randomised to receive either intramuscular ChAdOx1 nCoV-19 or a control vaccine (capsular group B meningococcal vaccine, 4CMenB),
Recruitment	The participants were recruited from COVID-19 vaccine trials conducted across several sites within the United Kingdom. They were randomised into vaccine groups.
Ethics oversight	The trials were conducted according to the principles of Good Clinical Practice and approved by the South Central Berkshire Research Ethics Committee (20/SC/0145, 20/SC/0179, 21/SC/0119, 21/SC/0022 and 21/SC/0054) and the UK regulatory agency (the Medicines and Healthcare products Regulatory Agency). We obtained informed consent from participants (or parent/guardians) for genetic data to be analysed, this was an optional consent in their clinical study consent forms.

Note that full information on the approval of the study protocol must also be provided in the manuscript.

Field-specific reporting

Please select the one below that is the best fit for your research. If you are not sure, read the appropriate sections before making your selection.

☒ Life sciences ☐ Behavioural & social sciences ☐ Ecological, evolutionary & environmental sciences

For a reference copy of the document with all sections, see nature.com/documents/nr-reporting-summary-flat.pdf

Life sciences study design

All studies must disclose on these points even when the disclosure is negative.

Sample size	No formal sample-size calculation were conducted for this genetic analysis, rather sample size was based on the opportunistic sample availability from COVID-19 vaccine trials (genetic analysis was an exploratory endpoint in these studies).
Data exclusions	Genetic samples were removed if they had high genotyping missingness, genetic sex did not match reported sex or they had high heterozygosity — these are metrics of poor sample or genotyping quality. Individuals were excluded if they had estimates ≥0.9 identity by descent, excluding the individual with the highest SNP missingness rate from each pair preferentially — to remove duplicated samples.
Replication	An independent cohort was used to replicate findings.
Randomization	Participants were randomised into vaccine groups.

Blinding	Participants were blinded to their vaccine allocation.

Reporting for specific materials, systems and methods

We require information from authors about some types of materials, experimental systems and methods used in many studies. Here, indicate whether each material, system or method listed is relevant to your study. If you are not sure if a list item applies to your research, read the appropriate section before selecting a response.

Materials & experimental systems

n/a	Involved in the study
☒	☐ Antibodies
☒	☐ Eukaryotic cell lines
☒	☐ Palaeontology and archaeology
☒	☐ Animals and other organisms
☐	☒ Clinical data
☒	☐ Dual use research of concern

Methods

n/a	Involved in the study
☒	☐ ChIP-seq
☒	☐ Flow cytometry
☒	☐ MRI-based neuroimaging

Clinical data

Policy information about clinical studies
All manuscripts should comply with the ICMJE guidelines for publication of clinical research and a completed CONSORT checklist must be included with all submissions.

Clinical trial registration	NCT04324606; NCT04400838; ISRCTN registry, 69254139 (EudraCT 2020–005085–33); ISRCTN Number: 27841311 (EudraCT Number: 2021-001275-16), ISRCTN Number: 15638344 (EudraCT number:2020-005765-13)
Study protocol	COV001 (https://clinicaltrials.gov/ct2/show/NCT04324606); COV002 (https://clinicaltrials.gov/ct2/show/NCT04400838); COMCOV (https://comcovstudy.org.uk/files/com-covprotocolv9220-sept-2021cleanpdf); COMCOV2 (https://comcovstudy.org.uk/files/com-cov2protocolv6122-sep-2021cleanpdf); COV006 (https://www.isrctn.com/ISRCTN15638344)
Data collection	The participants were recruited from COVID-19 vaccine trials conducted across several sites within the United Kingdom. Data between April 2020 and January 2022 were included in this manuscript.
Outcomes	Genetic analyses were included as exploratory outcome in the clinical trials from which these data were generated.
